# Cross-country health inequalities of four common nutritional deficiencies among children, 1990 to 2019: data from the Global Burden of Disease Study 2019

**DOI:** 10.1186/s12889-024-17942-y

**Published:** 2024-02-15

**Authors:** Wenkai Jiang, Xiao Li, Ruiying Wang, Yan Du, Wence Zhou

**Affiliations:** 1 The Second Clinical Medical College, Lanzhou University, No. 222 Tianshui Road (South), Cheng-Guan District, 730030 Lanzhou City, Gansu Province China; 2 The First Clinical Medical College, Lanzhou University, 730030 Lanzhou, China

**Keywords:** Health inequities, Child, Malnutrition, Global burden of Disease, Prevalence, Disability-adjusted life year

## Abstract

**Background:**

Nutritional deficiencies remain serious medical and public health issues worldwide, especially in children. This study aims to analyze cross-country inequality in four common nutritional deficiencies (protein-energy malnutrition, dietary iron deficiency, vitamin A deficiency and iodine deficiency) among children from 1990 to 2019 based on Global Burden of Disease (GBD) 2019 data.

**Methods:**

Prevalence and disability-adjusted life years (DALYs) data as measures of four nutritional deficiency burdens in people aged 0 to 14 years were extracted from the GBD Results Tool. We analyzed temporal trends in prevalence by calculating the average annual percent change (AAPC) and quantified cross-country inequalities in disease burden using the slope index.

**Results:**

Globally, the age-standardized prevalence rates of dietary iron deficiency, vitamin A deficiency and iodine deficiency decreased, with AAPCs of -0.14 (-0.15 to -0.12), -2.77 (-2.96 to -2.58), and -2.17 (-2.3 to -2.03) from 1999 to 2019, respectively. Significant reductions in socio-demographic index (SDI)-related inequality occurred in protein-energy malnutrition and vitamin A deficiency, while the health inequality for dietary iron deficiency and iodine deficiency remained basically unchanged. The age-standardized prevalence and DALY rates of the four nutritional deficiencies decreased as the SDI and healthcare access and quality index increased.

**Conclusions:**

The global burden of nutritional deficiency has decreased since 1990, but cross-country health inequalities still exist. More efficient public health measures are needed to reduce disease burdens, particularly in low-SDI countries/territories.

**Supplementary Information:**

The online version contains supplementary material available at 10.1186/s12889-024-17942-y.

## Introduction

Nutritional deficiencies significantly contribute to the global burden of disease. Common nutritional deficiencies include protein-energy malnutrition and micronutrient deficiencies (iron, vitamin A, and iodine). Protein-energy malnutrition is a prevalent issue worldwide, and there is a clear correlation between its burden and socioeconomic status [1]. Iron deficiency, which mainly leads to anemia, is one of the main contributors to the global burden of disease. Approximately 1.24 billion individuals experience iron deficiency anemia [2]. Vitamin A deficiency in children leads to stunted growth, vision loss and increased risk of infection [3]. Approximately 190 million preschool children are exposed to vitamin A deficiency worldwide [4]. Additionally, two billion people have insufficient iodine intake worldwide, with those in Africa and South Asia being particularly affected [5].

Childhood is a critical period for human growth, and malnutrition during this time can lead to wasting, underweight, and stunted growth, which can have a detrimental impact on children’s health. Nutrients, including proteins and microelements, contribute to the development of the motor system and nervous system in children. Health inequalities exist across different countries/territories, particularly for infectious and nutritional diseases [6]. Globally, one-third of children younger than 5 years old experience some form of malnutrition [7]. In low-income and middle-income regions, nutritional health has become increasingly unaffordable for millions of children. Children are often in a vulnerable position in the allocation of resources such as access to health care. They may face lower health coverage and lack the basic elements of a minimally sufficient health care system.

Universal health coverage is now an important goal of medical and public health. The World Health Organization proposed that universal health coverage policy aims to decrease inequalities and provide “health for all” [8]. One of the vital parts of “Countdown to 2030” aims to support the monitoring and measurement of children’s health [9]. Currently, children still face unhealthy diets and poverty [10–12]. Under these circumstances, additional attention should be given to the disease burden and health inequalities in nutritional health among children. In this study, we used data from the Global Burden of Disease 2019 (GBD 2019) to analyze the global, regional, and national prevalence of four common nutritional deficiencies among children, and to quantify cross-country inequalities, aiming to provide new insight into public health and children’s nutrition and health.

## Methods

### Data sources

Data were downloaded from GBD 2019 (https://vizhub.healthdata.org/gbd-results/). GBD 2019 estimated 369 diseases and injuries in seven GBD super-regions, 21 GBD regions and all countries/territories [13]. In this study, rates, numbers and their 95% uncertainty intervals (UIs) of prevalence and disability-adjusted life years (DALYs) were reported for both sexes, 3 age groups (GBD 2019 provided age groups in 5-year intervals: <5 years, 5 to 9 years, and 10 to 14 years) and 30 years (GBD 2019 provided data from 1990 to 2019). More information about GBD 2019 and data selection are provided in Additional File 1.

### Disease definitions

In the GBD 2019, we selected four common nutritional deficiencies: protein-energy malnutrition, dietary iron deficiency, vitamin A deficiency and iodine deficiency [13]. Protein-energy malnutrition encompasses moderate and severe acute malnutrition [13]. It is defined based on the weight-for-height Z-scores from the World Health Organization 2006 growth standard for children, including: moderate wasting without oedema (Z-scores < − 2 SD to < − 3 SD), moderate wasting with oedema (Z-scores < − 2 SD to < − 3 SD), severe wasting without oedema (Z-scores < − 3 SD) and severe wasting with oedema (Z-scores < -3SD) [13]. In GBD 2019, dietary iron deficiency was defined as insufficient iron intake to meet the body’s needs, excluding absolute or functional iron deficiency caused by other factors [13]. Vitamin A deficiency was defined as a prevalence of serum retinol < 0.7 µmol/L [13]. The non-fatal burden of iodine deficiency estimates only include the subset associated with visible goiter (grade 2) and its related consequences, such as thyroid dysfunction, heart failure, and intellectual disability. It does not encompass estimates of sub-clinical iodine deficiency or non-visible goiter (grade 1) caused by iodine deficiency [13]. The 10th revision of the International Classification of Diseases for four nutritional deficiencies includes E40-E46.9, E64.0, D50-D50.9, E50-E50.9, E64.1, and E00-E02.

### Socio-demographic index (SDI) and healthcare access and quality index (HAQ)

The SDI is a comprehensive index used to measure the social development level of a region or country/territory. It takes into account per capita income, average school years for individuals over 15 years old, and the total fertility rate for females aged 25 years or younger, which ranges from 0 to 1 [14]. Based on the SDI values, countries/territories are divided into five groups: high SDI (> 0.805), high-middle SDI (0.689 to 0.805), middle SDI (0.608 to 0.689), low-middle SDI (0.455 to 0.608) and low SDI (< 0.455). The SDI values for different countries/territories can be found in Additional File 2. The HAQ index measures mortality amenable to healthcare access and quality [15]. It was developed by the GBD study group and is based on risk, age and population-standardized cause-specific death rates of 32 causes of morality amenable to personal healthcare. The HAQ index provides a summary measure on a scale of 0 to 100 to facilitate comparison of personal health-care access and quality by geography [16].

### Data analysis

All statistical analyses were performed using R Studio software (version 4.2.2) and Joinpoint Software (version 5.0.2, National Cancer Institute, USA). In the GBD Study, one DALY is interpreted as one lost year of healthy life, and can be calculated by summing the year of life lost (healthy time lost due to premature mortality) and the year lived with disability (healthy time lost due to living with disease or injury) [17]. The age-standardized rates (ASRs) and 95% confidence intervals (CIs) were calculated based on the world standard population reported in the GBD 2019, including age-standardized prevalence rate (ASPR, total cases per 100,000 population after age standardization) and age-standardized DALY rate [18]. The average annual percent changes (AAPCs) were calculated in Joinpoint model to analyze the temporal trends of ASRs [19]. The AAPC was calculated as a geometrically weighted average of annual percent change in Joinpoint regression analysis [20]. The AAPC and its 95% CI over 0 indicate an upward trend, while the AAPC and its 95% CI less than 0 indicate a downward trend [21].

The slope index of inequality is an absolute measure of inequality provided by the World Health Organization (WHO) Health Equity Assessment Toolkit Plus. The slope index of inequality was calculated by regressing DALY rates on an SDI-related relative social position scale, which was defined by the midpoint of the cumulative class interval of the population ranked by the SDI [22, 23]. In this study, the slope index of inequality represents the difference in estimated DALY between the country/territory with the lowest SDI and highest SDI, while taking into account the population size of the countries/territories using an appropriate regression model. To calculate the slope index, a weighted sample of the whole population is ranked from the country with the lowest SDI country/territory to the country/territory with the highest SDI. This ranking is weighted, accounting for the proportional distribution of the population within each subgroup. Greater absolute values of the slope index of inequality indicate higher levels of inequality. We also used scatter diagram and smoothing spline models to test for correlations between ASRs in 2019 and SDI and HAQ index values using the “ggplot2” package in R Studio software. All rates are shown per 100,000 persons.

## Results

### Protein-energy malnutrition

**Fig. 1:**
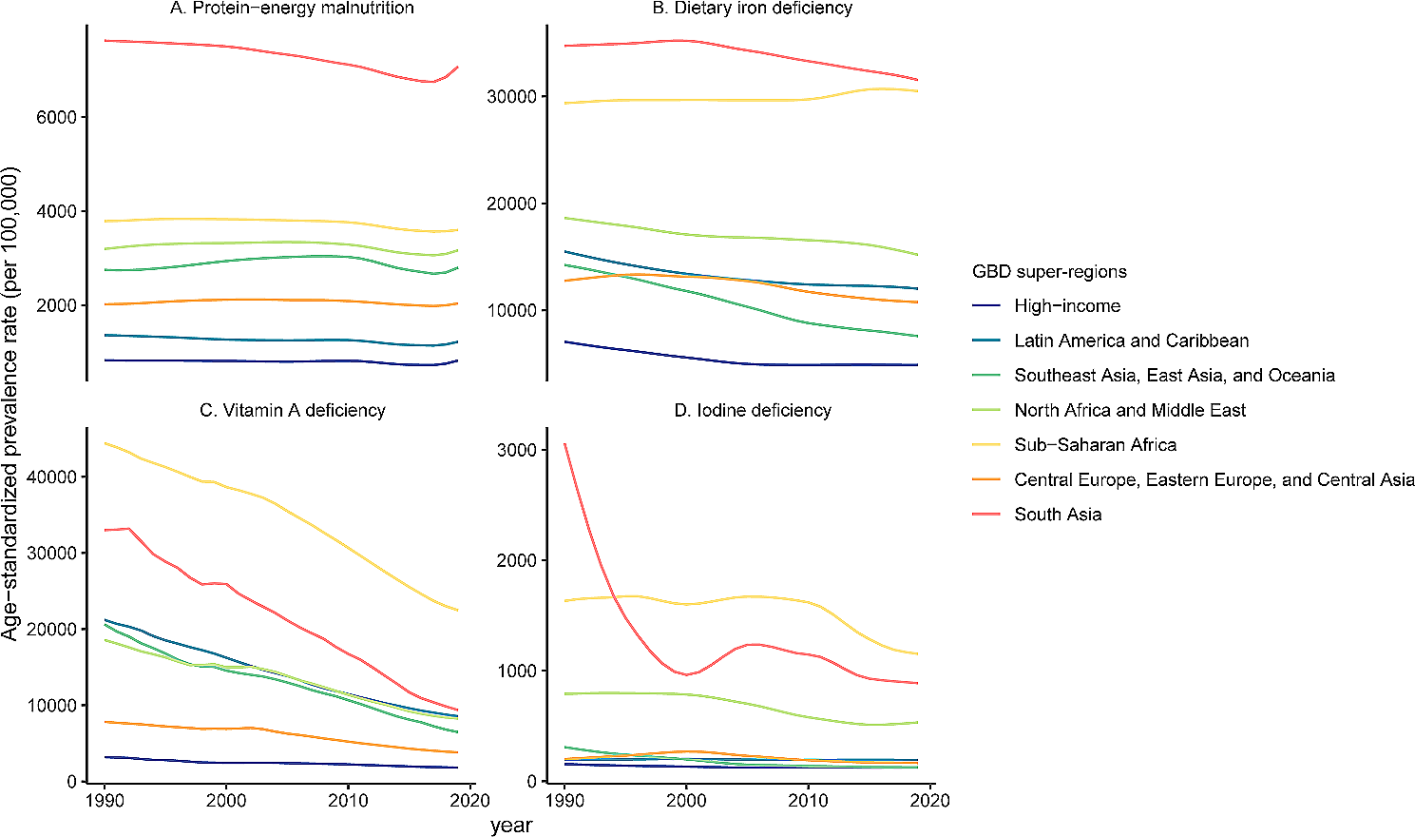
The ASPR of protein-energy malnutrition, dietary iron deficiency, vitamin A deficiency and iodine deficiency from 1990 to 2019 by GBD super-region

**Fig. 2:**
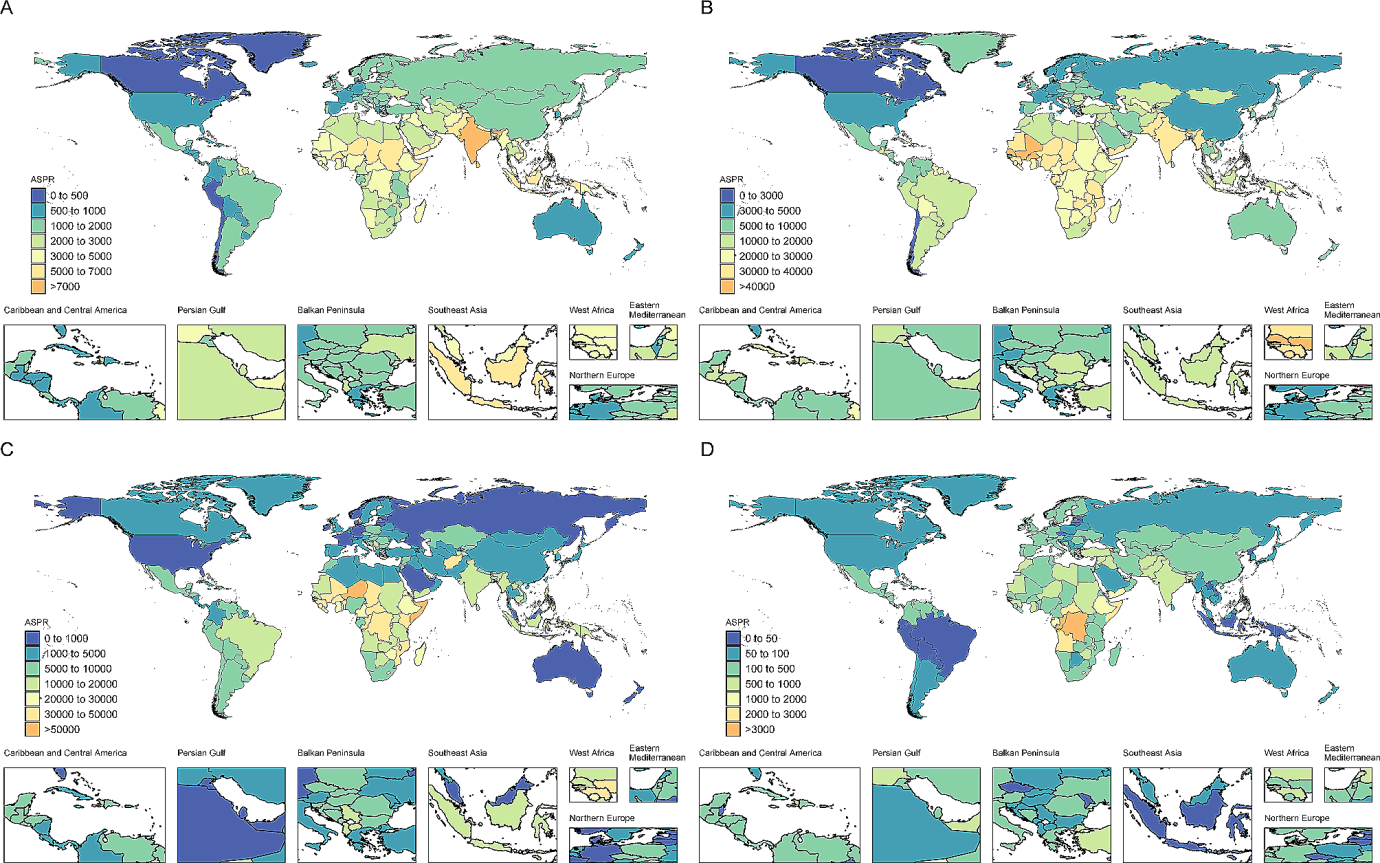
The ASPR of protein-energy malnutrition, dietary iron deficiency, vitamin A deficiency and iodine deficiency among all countries/territories in 2019

The global ASPR for protein-energy malnutrition among children did not significantly change from 1990 to 2019 (AAPC= -0.04, 95% CI: -0.12 to 0.04, Table 1). Figure 1A and Figure S1A display the temporal trends of the ASPR for protein-energy malnutrition at the global level and in seven GBD super-regions over the past 30 years. In 2019, there were 72,727,766 (68,933,020 to 77,526,486) cases of protein-energy malnutrition among children worldwide, which was 1.05 times greater than that in 1990. The highest proportion of prevalence cases was observed in children under the age of 5 (Figure S2). South Asia had the highest prevalence of cases (34,174,598) and ASPR (7069.8 per 100,000) in 2019 (Table S1, Figure S3). At the national level, India had the highest ASPR (7857.9 per 100,000), followed by Sri Lanka (7133.0 per 100,000) and Maldives (6917.7 per 100,000) (Fig. 2A). The pattern and trends of the age-standardized DALY rates were similar to those of the ASPR (Figure S4A, Table S2).

**Table 1:** Prevalence and its change trend of four common nutritional deficiencies among children in five SDI quintiles, 1990 to 2019

	Protein-energy malnutrition	Dietary iron deficiency	Vitamin A deficiency	Iodine deficiency
Global				
Number of prevalence in 2019 (million)	72.7 (68.9-77.5)	391.5 (382.8-400.5)	209.7 (196.2-225.1)	12.1 (8.1-17.1)
Percentage change, 1990 to 2019 (%)	5.1% (3 to 7.5%)	5.5% (2.7 to 8.5%)	-44.1% (-48.2% to -39.4%)	-38.3% (-44.6% to -32.5%)
ASPR in 2019 (per 100,000)	3796.01 (3584.19 to 4063.58)	20146.35 (19407.85 to 20888.54)	10779.02 (9727.6 to 12133.06)	604.14 (403.46 to 856.81)
AAPC, 1990 to 2019	-0.04 (-0.12 to 0.04)	-0.14 (-0.15 to -0.12)	-2.77 (-2.96 to -2.58)	-2.17 (-2.3 to -2.03)
High SDI				
Number of prevalence in 2019 (million)	1.5 (1.3 to 1.8)	7.4 (6.5-8.4)	1.7 (1.5-1.9)	0.2 (0.1-0.3)
Percentage change, 1990 to 2019	-5.9% (-9.4% to -2.3%)	-36.6% (-45.3% to -26.3%)	-67.4% (-72.5% to -60.8%)	-11.5% (-13.8% to -8.9%)
ASPR in 2019 (per 100,000)	926.07 (782.3 to 1108.86)	4671.08 (3765.88 to 5786.17)	1030.23 (848.47 to 1263.04)	104.2 (61.53 to 160.08)
AAPC, 1990 to 2019	0 (-0.09 to 0.09)	-1.3 (-1.35 to -1.25)	-2.78 (-2.92 to -2.64)	-0.3 (-0.35 to -0.25)
High-middle SDI				
Number of prevalence in 2019 (million)	5.8 (5.3-6.5)	22.4 (21-23.9)	8.7 (7.8-9.6)	0.6 (0.4-0.8)
Percentage change, 1990 to 2019	-13.1% (-16.5% to -9.4%)	-45.7% (-49.7% to -41.3%)	-72.9% (-76.3% to -68.8%)	-57.5% (-61.5% to -53.3%)
ASPR in 2019 (per 100,000)	2432.83 (2197.52 to 2729.05)	9271.51 (8331.91 to 10291.1)	3558.31 (2988.12 to 4252.64)	224.84 (144.32 to 333.17)
AAPC, 1990 to 2019	0.28 (0.21 to 0.34)	-1.34 (-1.37 to -1.31)	-3.8 (-3.91 to -3.69)	-2.24 (-2.34 to -2.15)
Middle SDI				
Number of prevalence in 2019 (million)	17.7 (16.4-19.2)	74.9 (71.8-77.9)	32.4 (29.3-35.7)	1.7 (1.1-2.5))
Percentage change, 1990 to 2019	-5.7% (-8.8% to -2.4%)	-28.6% (-32.2% to -24.9%)	-69.2% (-72.8% to -65.3%)	-55% (-59% to -51.1%)
ASPR in 2019 (per 100,000)	3292.9 (3049.68 to 3607.76)	13720.54 (12825.23 to 14632.6)	5905.03 (4942.86 to 7035.82)	297.54 (191.98 to 438.62)
AAPC, 1990 to 2019	0.09 (0.04 to 0.14)	-0.93 (-0.95 to -0.92)	-4.14 (-4.25 to -4.03)	-2.68 (-2.86 to -2.49)
Low-middle SDI				
Number of prevalence in 2019 (million)	25.1 (23.9-26.6)	131.6 (126.7-136.9)	60.1 (53.3-68.3)	3.8 (2.5-5.5)
Percentage change, 1990 to 2019	-9% (-11.4% to -6.4%)	0.4% (-3.9 to 5.1%)	-57.5% (-62.4% to -51.1%)	-60.6% (-66.1% to -55.1%)
ASPR in 2019 (per 100,000)	5009.55 (4734.76 to 5340.25)	25497.06 (24067.64 to 27050.28)	11631.03 (9576.04 to 14527.17)	690.11 (450.38 to 1000.23)
AAPC, 1990 to 2019	-0.51 (-0.54 to -0.48)	-0.38 (-0.39 to -0.36)	-3.76 (-3.99 to -3.53)	-3.89 (-4.12 to -3.66)
Low SDI				
Number of prevalence in 2019 (million)	22.6 (21.9-23.4)	154.9 (150.6-159.2)	106.7 (100.5-113.4)	5.9 (4-8.2)
Percentage change, 1990 to 2019	55.6% (53 to 58.3%)	89.1% (82.3 to 95.7%)	2.6% (-4.5 to 9.5%)	23.9% (10.2 to 35%)
ASPR in 2019 (per 100,000)	4641.11 (4457.26 to 4858.39)	32516.96 (30993.53 to 34036.61)	22389.56 (20252.91 to 24796.64)	1275.4 (861.44 to 1762.91)
AAPC, 1990 to 2019	-0.49 (-0.52 to -0.47)	-0.04 (-0.06 to -0.02)	-2.41 (-2.58 to -2.25)	-1.8 (-1.89 to -1.71)

SDI: socio-demographic index; ASPR: age-standardized prevalence rate; AAPC: average annual percent change

**Fig. 3:**
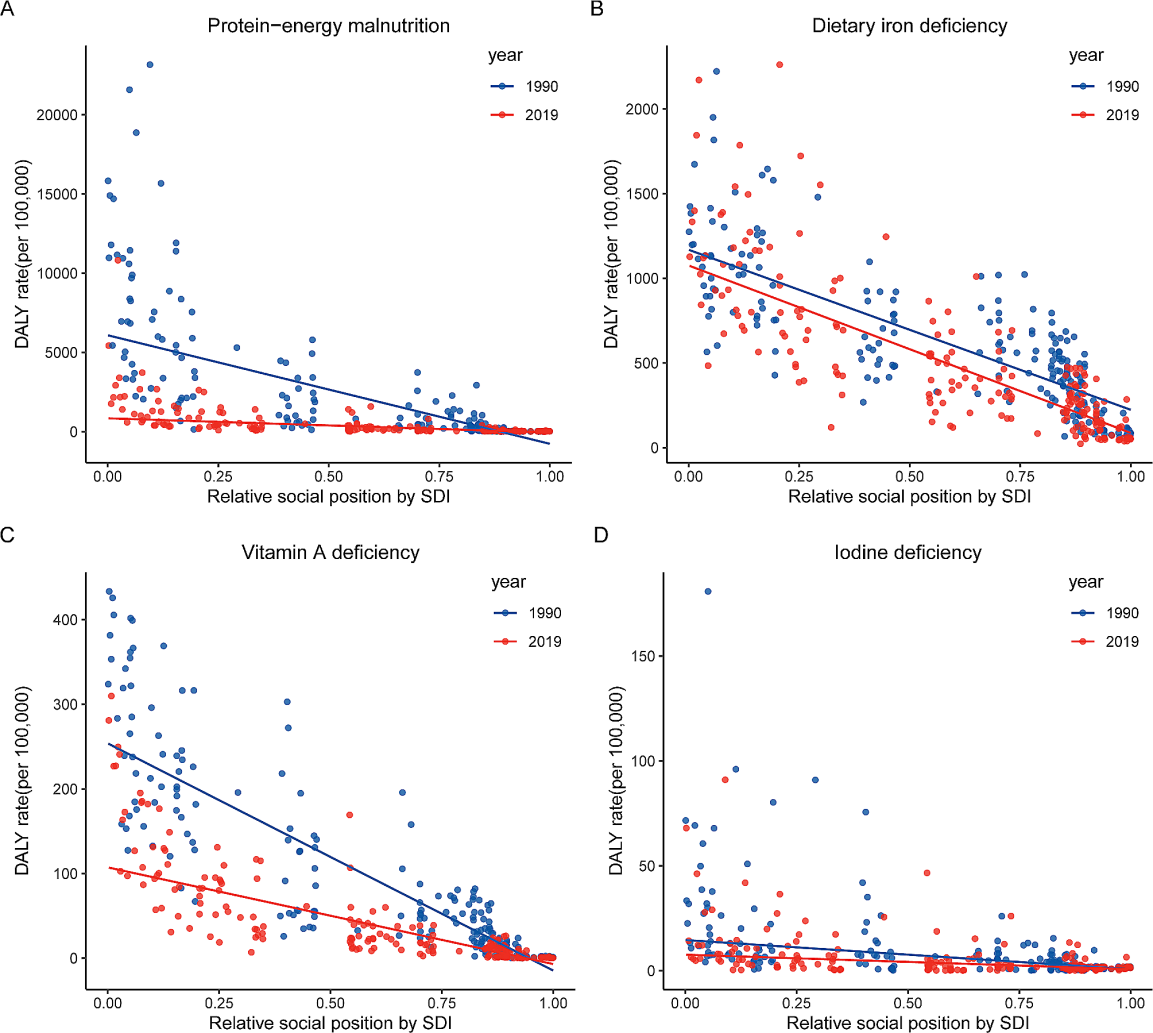
SDI-related health inequality regression for the burden of DALY due to four nutritional deficiencies, 1990 and 2019

Significant SDI-related inequalities in the burden of protein-energy malnutrition were observed. As shown in Fig. 3A, the gap in DALY rates between the highest and lowest SDI countries decreased significantly, with a slope index changing from -6851.5 (95% CI: -7367.3 to -6335.8) in 1990 to -907.4 (95% CI: -988.2 to -826.6) in 2019, indicating a reduction in health inequality.

### Dietary iron deficiency

The ASPR of dietary iron deficiency decreased worldwide, with an AAPC of -0.14 (95% CI: -0.15 to -0.12) from 1990 to 2019. Figure 1B and Figure S1B show the temporal trends of the ASPR for dietary iron deficiency at the global level and in seven GBD super-regions during the past 30 years. The prevalence of cases slightly increased, with a percentage change of 5.5% (2.7 to 8.5%) during the past three decades. Children under the age of 5 accounted for nearly half (Figure S2). Among the 21 GBD regions, the highest ASPRs were observed in Western Sub-Saharan Africa and South Asia, with values of 36715.5 per 100,000 and 31523.9 per 100,000, respectively (Table S1, Figure S5). At the national level, the ASPR was highest in Bhutan, followed by Mali and Gambia (Fig. 2B). The pattern and trends of the age-standardized DALY rates were similar to those of the ASPR (Figure S4B, Table S2).

Significant SDI-related inequalities in the burden of protein-energy malnutrition were observed. As shown in Fig. 3B, the gap in the DALY rate between the highest and lowest SDI countries slightly increased, with a slope index from -943.1 (95% CI: -1033.5 to -852.8) in 1990 to -992.5 (95% CI: -1080.1 to -904.8) in 2019.

### Vitamin A deficiency

The global ASPR of vitamin A deficiency showed a downward trend over the past 30 years (AAPC= -2.77, 95% CI: -2.96 to -2.58). The ASPRs in seven GBD super-regions all showed downward trends (Fig. 1C). There was a 44% (39 to 48%) reduction in the prevalence of cases of vitamin A deficiency among children from 1990 to 2019. Children under the age of 5 accounted for nearly half of the prevalence cases worldwide (Figure S2). At the regional level, the top three regions with the highest ASPR of vitamin A deficiency were observed in Sub-Saharan Africa (including Central, Western and Eastern) in 2019 (Table S1, Figure S6). Somalia (64133.5 per 100,000), Niger (50833.9 per 100,000) and Chad (39742.5 per 100,000) are the top three countries with the highest ASPRs in 2019 (Fig. 2C). Trends in the age-standardized DALY rates were similar to ASPR (Figure S4C, Table S2).

Significant SDI-related inequalities in the burden of protein-energy malnutrition were observed. As shown in Fig. 3C, the slope index of inequality decreased from -268.5 (95% CI: -283.8 to -253.2) in 1990 to -115.0 (95% CI: -122.5 to -107.6) in 2019.

### Iodine deficiency

The global ASPR of iodine deficiency showed a downward trend, with an AAPC of -2.17 (95% CI: -2.3 to -2.03). Figure 1D and Figure S1D show the temporal trends of the ASPR for iodine deficiency at the global level and in seven GBD super-regions during the past 30 years. Moreover, the global prevalence of iodine deficiency also significantly decreased, from 19,635,387 in 1990 to 12,110,179 in 2019 (percentage change: -38%, 95% UI: -45% to -33%). Children aged 10 to 14 years had the highest prevalence (Figure S2). Among the 21 GBD regions, the highest ASPR was observed in Central Sub-Saharan Africa ( 5478.6 per 100,000), which was much higher than that in other regions (Table 1, Figure S7). As shown in Fig. 2D, Democratic Republic of the Congo (6808.4 per 100,000) had the highest ASPR in 2019. Trends in the age-standardized DALY rates were similar to those in the ASPR for iodine deficiency (Figure S4D, Table S2).

As illustrated by the slope index of inequality, the gap in the DALY rate between the highest and lowest SDI countries significantly decreased, from  -13.9 (95% CI: -15.8 to -12.0) in 1990 to -6.9 (95% CI: -8.1 to -5.7) in 2019 (Fig. 3D).

### Burdens by SDI quintiles and HAQ score

From 1990 to 2019, the highest disease burden of the four nutritional deficiencies was observed in the low-SDI region (Figures S8 and S9). In 2019, the ASPRs ranged from 5009.5 per 100,000 in the low-SDI quintile to 926.1 per 100,000 in the high-SDI quintile for protein-energy malnutrition, from 32517.0 per 100,000 in the low-SDI quintile to 4671.1 per 100,000 in the high-SDI quintile for dietary iron deficiency, from 22389.6 per 100,000 in the low-SDI quintile to 1030.2 per 100,000 in the high-SDI quintile for vitamin A deficiency, and from 1275.4 per 100,000 in the low-SDI quintile to 104.2 per 100,000 in the high-SDI quintile for iodine deficiency.

**Fig. 4:**
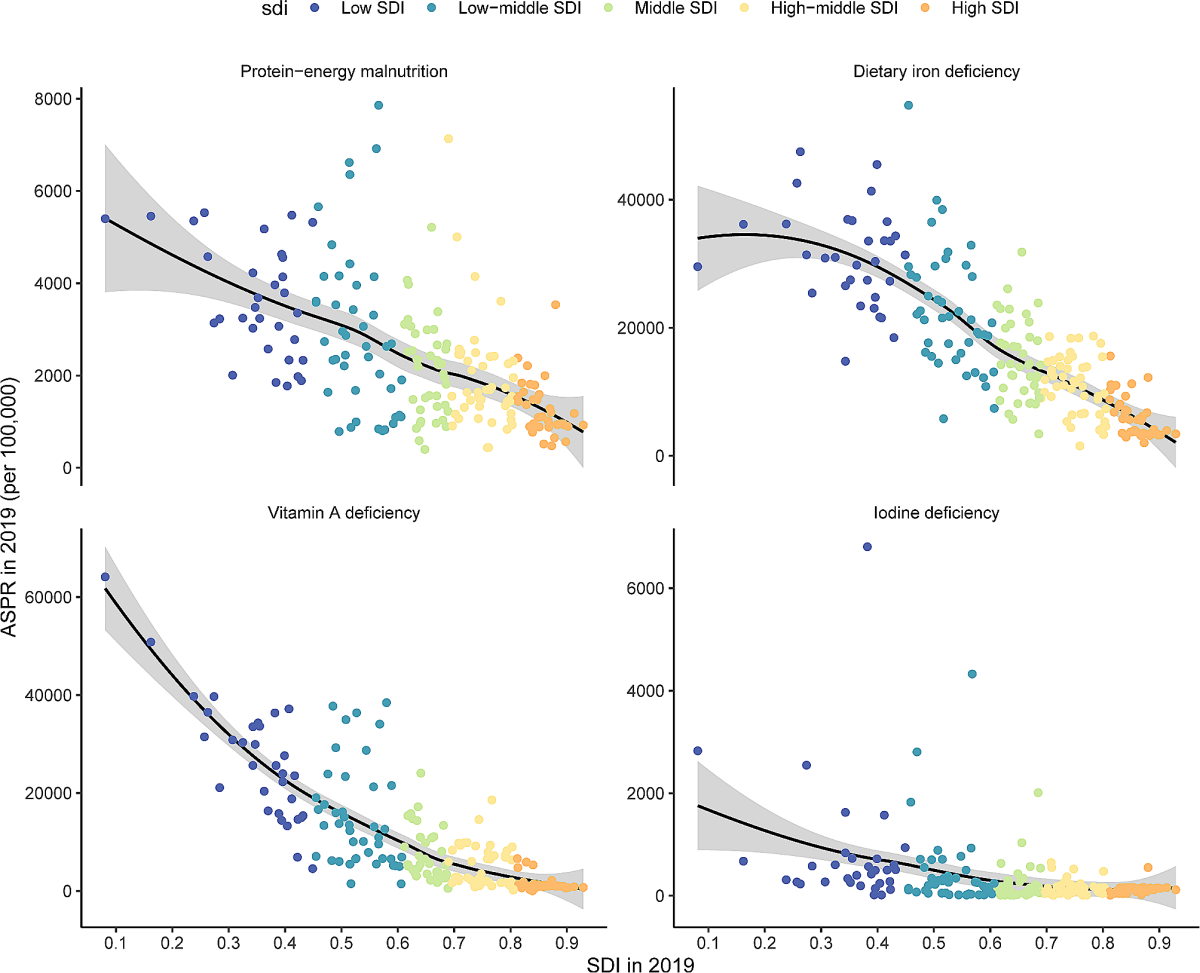
The relationships between ASPRs of four nutritional deficiencies and SDI across all countries/territories in 2019

**Fig. 5:**
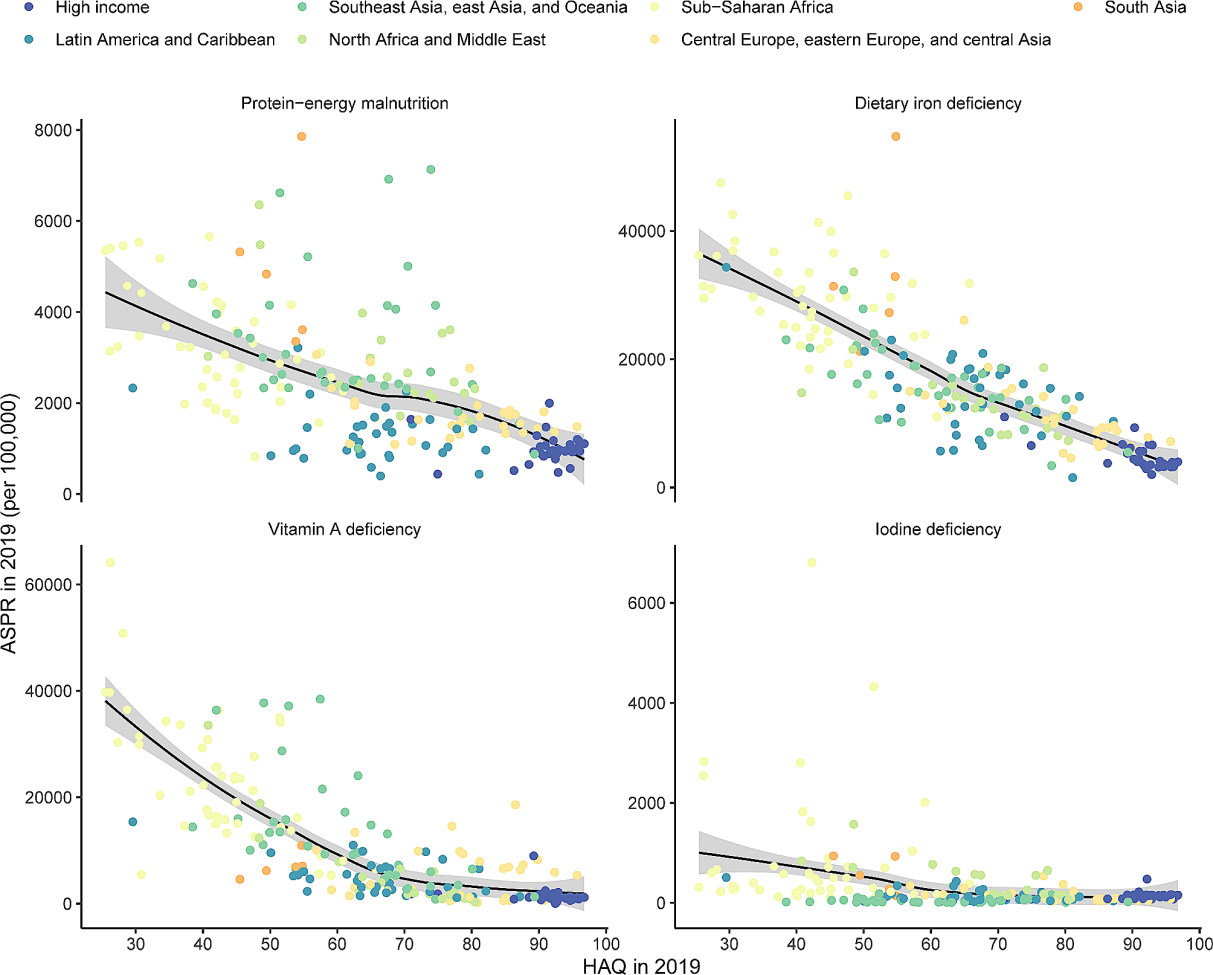
The relationships between ASPRs of four nutritional deficiencies and HAQ across all countries/territories in 2019

The relationships between the ASPR (in 2019) and the HAQ and SDI (in 2019) among all countries/territories are shown in Figs. 4 and 5. The results showed that as the SDI and HAQ values increased, the ASPRs of the four nutritional deficiencies decreased. The age-standardized DALY rates of four nutritional deficiencies showed similar patterns (Figure S10 and S11). Similar to the national level, the age-standardized prevalence and DALY rates decreased with increasing SDI during the past 30 years in 21 GBD regions (Figures S12 and S13).

## Discussion

This analysis provides estimates of trends in the prevalence and DALYs of four common nutritional deficiencies among children in all countries/territories from 1990 to 2019. Our results showed that age-standardized prevalence and DALY rates have declined worldwide over the past three decades. Cross-country health inequalities for dietary iron deficiency and iodine deficiency remained basically unchanged. We also found that nutritional deficiencies are serious medical problems in Sub-Saharan Africa and South Asia, with high prevalence and DALYs, while countries/territories with high SDI not. This suggests that we should pay more attention to nutritional health, children’s health and health coverage in low-income countries/territories.

The global age-standardized prevalence and DALY rates of dietary iron deficiency, vitamin A deficiency and iodine deficiency showed downward trends, mainly due to the increase in the consumption of animal products, dietary diversification and improved access to microelements [24]. Moreover, continuous improvements in the global economy, the quality of health care, and food supplementation strategies contributed to these reductions. However, countries/territories in Sub-Saharan Africa and South Asia suffer more from nutritional deficiencies among children. This finding is consistent with the results of the GBD Study 2015 [25]. These differences may be associated with geographical, cultural, economic, and demographic factors. In Sub-Saharan Africa, rapid population growth, inefficient agriculture and industry, and inadequate health infrastructure lead to malnutrition and disease [26]. Inadequate food supply, low household income and inappropriate child’s care cause malnutrition in developing South Asian countries [27]. Moreover, low consumption of red or processed meats and low intake of other healthy components (fruit, non-starchy vegetables and legumes/nuts) in South Asia and Sub-Saharan Africa caused regional differences [28].

One possible explanation for health inequality among children is that countries/territories with low SDI have not received significant development assistance for health [25]. Moreover, we analyzed the health inequalities of nutritional deficiencies among children to determine the disparities in child medical care and nutritional health among countries/territories. There were reductions in the SDI-related DALY rates of protein-energy malnutrition and vitamin A deficiency, which means that the global control and prevention of nutritional diseases among children has partially improved over the past 30 years, and may provide references for eliminating health inequities caused by other diseases in children.

Protein is one of the three major nutrients [29]. The recommended dietary protein intake for a healthy adult with minimal physical activity is 0.8 g per kilogram body weight per day, and this number is greater for infants and children because they grow and gain protein [29]. Insufficient protein nutrition usually manifests as marasmus (a general wasting caused by protein and energy deficiency) and malnutrition (characterized by significant edema and a lack of protein quantity and quality), which may lead to stunting, physical weakness, edema, and impaired immunity [30]. Some African and South Asian countries/territories have a higher prevalence of protein energy malnutrition, which is consistent with our results [31]. In these countries/territories, only 3% of total dietary energy is derived from meat and animal offal, leading to serious inadequate animal protein intake, and dietary protein consumption per day in people in Africa far below the world average level [32]. Moreover, maize is a staple food in Sub-Saharan countries/territories. Although maize provides human macro- and micronutrients, it lacks adequate amounts of essential amino acids such as lysine and tryptophan. People who derive more than 50% of their daily energy intake from maize may suffer from protein-energy malnutrition [33].

Iron is an important microelement for the neonate, and in early childhood [34]. During the past few years, iron deficiency has become more common in disadvantaged subpopulations, such as people in low- and middle-income countries/territories [35]. Approximately 40% of children aged 1 to 3 years have iron deficiency worldwide, and approximately 70% in Central and West Africa [34]. In childhood, iron deficiency may negatively affect neurodevelopment and exercise capacity, and may increase susceptibility to infection [34]. Additionally, low iron supplementation in pregnant women may lead to a high prevalence of dietary iron deficiency among children aged under five years. One of the WHO’s goals for global health is to achieve a 50% reduction in anemia prevalence in females by 2025 [35]. Therefore, we should not only pay attention to iron deficiency in children, but also to iron deficiency in females, especially pregnant females.

Vitamin A deficiency is a considerable nutritional problem in the developing world, especially affecting the health of infants and young children [36]. Approximately 127 million preschool-aged children are vitamin A deficient [36]. This can be attributed to the lack of dietary diversity and improved access to vitamin-rich foods in low-income countries/territories. Vitamin A supplementation is one of the current public health interventions and affects approximately 250 million children every year [37]. Significant reductions in child mortality in clinical trials of vitamin A supplements have led to the use of vitamin A supplements in low- and middle-income countries/territories. A meta-analysis of 215,633 children showed that vitamin A supplementation could reduce the prevalence of diarrhea, measles, night blindness and xerophthalmia, and all-cause mortality [38]. Despite the fact that more than two-thirds of children in developing countries/territories take vitamin A supplements, the prevalence of vitamin A deficiency has not changed obviously [39]. This emphasizes the need for better methods and policies to reduce the prevalence of vitamin A deficiency among children in high burden countries/territories.

Iodine is essential for normal growth and neurodevelopment in infants and young children [40]. The intelligence quotient levels of children living in areas with severe iodine deficiency were an average of 6 to 12 points lower than that of children living in areas with sufficient iodine [40]. Between 2005 and 2020, the median urinary iodine concentration in pregnancy in African countries was still lower than 150 µg/l [41]. The World Summit for Children at the United Nations in 1990 endorsed the goal of eliminating iodine deficiency as a public health problem by the year 2000 [42]. There have been several achievements in the prevention of iodine deficiency: 68% of households in 130 countries with iodine deficiency as a public health problem had access to iodized salt in 1999, compared to less than 10% in 1990 [43]. Global urinary iodine concentration survey in 2014 showed that during the past decade, the number of iodine-sufficient countries increased from 67 to 112, indicating great progress [44]. However, many countries/territories lack monitoring of the ongoing programs for iodine deficiency disease, especially national urinary iodine concentration surveys in pregnant women. China is one of the countries that has sufficient experience monitoring and eliminating iodine deficiency. Since 1995, all provinces in China have adopted universal salt iodization as the main measure for preventing iodine deficiency [42].

We found that the disease burdens of four nutritional deficiencies among children were negatively correlated with the SDI and HAQ scores, which may be explained by the influence of inadequate dietary diversity and poor life conditions. Currently, economic downturns and population pressure may have some negative impacts on the global strategy for improving children’s nutritional status. Countries/territories in the low and low-middle SDI quintiles are disadvantaged in terms of social development and medical care. Food and nutritional supplements in many countries/territories still fall far short of people’s needs. Unemployment, low income and population growth exacerbate the nutritional health crisis in low-income countries/territories, and children are the most affected in this process. A growing population size will increase nutritional, educational and medical needs, especially for children. Moreover, pregnant women in low-income countries/territories are also affected by nutritional deficiencies, such as dietary iron deficiency and iodine deficiency, which may further affect the growth of fetuses and newborns. In contrast, in addition to completing health care and food safety, people in high-income countries/territories tend to receive better nutrition and health education [45].

Under these circumstances, medical policies should give immediate priority to children, and we should prioritize health equity for children within and across countries first. Monitoring of nutritional disease prevalence, increased investment school-based health promotion, and primary health-care services are required in developing countries/territories. For instance, the Indian Government developed a National Non-Communicable Diseases Monitoring Framework and Action Plan 2014 to promote youth health and wellbeing [46]. The Global Alliance for Improved Nutrition provided projects in 14 countries to increase dietary iron intake, including iron fortification of fish sauce in Vietnam and wheat and maize flour in South Africa [47]. In addition, we should improve dietary quality and promote healthy eating habits in early childhood. Increasing intakes of fruits, vegetables, and plant oils will have the largest impact on dietary quality of children in these countries. We also need to face the challenge of improving the nutritional health of children, such as the effects of natural disasters and infectious diseases on children’s healthy diets.

The following limitations should be considered when interpreting the results of this study. First, GBD estimates were limited by the quality of each country’s disease registration databases. Accurate and adequate national data on the prevalence of disease were available from high-income countries/territories. For some low-income countries/territories, there are insufficient diagnostic and mortality data in the registration systems, and GBD estimates relied on modeling (DisMod-MR) [13]. Thus, the prevalence or DALYs may be underestimated in some countries/territories with low or low-middle SDI values, leading to inaccurate data in these countries/territories. Moreover, GBD 2019 only included data for protein-energy malnutrition, dietary iron deficiency, vitamin A deficiency and iodine deficiency. There was a lack of adequate data on other nutritional deficiencies, such as vitamin C and folate deficiencies. Future work should focus on more primary data on nutritional deficiency incidence, prevalence, mortality and DALYs. More public health support should be given to individuals in low-income countries/territories to obtain more accurate epidemiological data, such as by establishing registration databases or conducting longer-term cohort studies. The economic burden caused by children’s nutritional deficiencies should also be given more attention.

## Conclusion

The global burden of nutritional deficiencies among children has decreased since 1990, but the prevalence of protein-energy malnutrition in South Asia and micronutrient deficiencies in Sub-Saharan Africa are still high. Most importantly, cross-country health inequalities still exist due to differences in social development among countries/territories. To reduce health inequalities and achieve universal health coverage, we need to pay more attention to children’s nutritional health, improve dietary quality, and increase medical support for low-income countries/territories.

### Electronic supplementary material

Below is the link to the electronic supplementary material.

Supplementary Material 1

## Data Availability

The datasets generated and/or analysed during the current study are available in the the Global Health Data Exchange (http://ghdx.healthdata.org/gbd-results-tool).

## Abbreviations

AAPC: Average annual percent change
ASR: Age-standardized rate
ASPR: Age-standardized prevalence rate
DALY: Disability-adjusted life year
GBD: Global Burden of Disease
HAQ: Healthcare Access and Quality
SDI: Socio-demographic index
WHO: World Health Organization
