# Supplementary material for: Cross-country health inequalities of four common nutritional deficiencies among children, 1990 to 2019: data from the Global Burden of Disease Study 2019
Source: BMC Public Health. 2024 Feb 15;24:486. doi: 10.1186/s12889-024-17942-y (PMC10870451; doi:10.1186/s12889-024-17942-y)
Supplement: Supplementary file 1 — Supplementary Material 1 [file 12889_2024_17942_MOESM1_ESM.pdf]

Additional files

Additional File 1: Overview of Global Burden of Disease Study

Additional File 2: Socio-demographic index of all countries/territories in 2019

Additional File 3: Supplementary figures

Figure S1 Global age-standardized prevalence rates of protein-energy malnutrition, dietary iron deficiency, vitamin A deficiency and iodine deficiency in males and females, 1990 to 2019

Figure S2 Temporal change in the relative proportion of four nutritional deficiencies prevalence cases across age groups, 1990 to 2019.

Figure S3 The age-standardized prevalence and DALY rates of protein-energy malnutrition by sex in 21 GBD regions, 2019

Figure S4 Global age-standardized DALY rates of protein-energy malnutrition, dietary iron deficiency, vitamin A deficiency and iodine deficiency in males and females, 1990 to 2019

Figure S5 The age-standardized prevalence and DALY rates of dietary iron deficiency by sex in 21 GBD regions, 2019.

Figure S6 The age-standardized prevalence and DALY rates of vitamin A deficiency by sex in 21 GBD regions, 2019.

Figure S7 The age-standardized prevalence and DALY rates of iodine deficiency by sex in 21 GBD regions, 2019.

Figure S8 The age-standardized prevalence rates of protein-energy malnutrition, dietary iron deficiency, vitamin A deficiency and iodine deficiency in different SDI quintiles, 1990 to 2019

Figure S9 The age-standardized DALY rates of protein-energy malnutrition, dietary iron deficiency, vitamin A deficiency and iodine deficiency in different SDI quintiles, 1990 to 2019

Figure S10 The relationships between age-standardized DALY rates of four nutritional deficiencies and SDI across all countries/territories in 2019.

Figure S11 The relationships between age-standardized DALY rates of four nutritional deficiencies and HAQ across all countries/territories in 2019.

Figure S12 The age-standardized prevalence rates of protein-energy malnutrition, dietary iron deficiency, vitamin A deficiency and iodine deficiency for 21 GBD regions by socio-demographic index, 1990 to 2019

Figure S13 The age-standardized DALY rates of protein-energy malnutrition, dietary iron deficiency, vitamin A deficiency and iodine deficiency for 21 GBD regions by socio-demographic index, 1990 to 2019

Additional File 4: Supplementary tables

Table S1 Count and rates of protein-energy malnutrition, dietary iron deficiency, vitamin A deficiency and iodine deficiency prevalence in 21 GBD regions in 2019

Table S2 Count and rates of protein-energy malnutrition, dietary iron deficiency, vitamin A deficiency and iodine deficiency DALY in 21 GBD regions in 2019

Additional File 1: Overview of Global Burden of Disease Study

1. Overview

The Global Burden of Diseases Study (GBD) provides a systematic scientific assessment of published, publicly available, and contributed data on disease and injury incidence, prevalence, and mortality of diseases and injuries. It produced estimates for 204 countries and territories that were grouped into 21 GBD regions and seven super-regions [1].

2. Data

The GBD estimation process is based on identifying multiple relevant data sources for each disease or injury including censuses, household surveys, civil registration and vital statistics, disease registries, health service use, air pollution monitors, satellite imaging, disease notifications, and other sources [1]. For some diseases and injuries, processed data are modelled using standardized tools to generate estimates of each quantity of interest by age, sex, location, and year. DisMod-MR is a Bayesian meta-regression tool that allows evaluation of all available data on incidence, prevalence, remission, and mortality for a disease, enforcing consistency between epidemiological parameters [1]. The metric definitions are listed as follows

	Number	Rate
Prevalence	Total number of cases in the population	Total cases per 100,000 population
Disability adjusted life years (DALYs)	Number of DALYs in the population	DALYs per 100,000 population

3. Data selecting

The GBD 2019 cause list is organized into a hierarchy (four levels). Level 1 contains 3 groups, named communicable, maternal, neonatal and nutritional diseases,

noncommunicable diseases and injuries. In this study, the broad group “Nutritional deficiencies is at Level 2 under the Level 1 group “Communicable, maternal, neonatal and nutritional diseases”. GBD 2019 classified all nutritional deficiencies into five types according to the International Statistical Classification of Diseases and Related Health Problems, Tenth Revision. In this study, we selected the disease named “Iodine deficiency”, “Vitamin A deficiency”, “Dietary iron deficiency”, and “Protein-energy malnutrition”, which coded E40-E46.9, E64.0, D50-D50.9, E50-E50.9, E64.1, and E00-E02.

Reference

[1] GBD 2019 Diseases and Injuries Collaborators. Global burden of 369 diseases and injuries in 204 countries and territories, 1990-2019: a systematic analysis for the Global Burden of Disease Study 2019. Lancet. 2020; 396(10258): 1204-1222.

Additional File 2: Socio-demographic index of all countries/territories in 2019

Location	Year	SDI
Afghanistan	2019	0.343
Albania	2019	0.681
Algeria	2019	0.652
American Samoa	2019	0.712
Andorra	2019	0.894
Angola	2019	0.47
Antigua and Barbuda	2019	0.743
Argentina	2019	0.708
Armenia	2019	0.689
Australia	2019	0.839
Austria	2019	0.849
Azerbaijan	2019	0.683
Bahamas	2019	0.796
Bahrain	2019	0.751
Bangladesh	2019	0.483
Barbados	2019	0.742
Belarus	2019	0.745
Belgium	2019	0.851
Belize	2019	0.603
Benin	2019	0.352
Bermuda	2019	0.813
Bhutan	2019	0.455
Bolivia (Plurinational State of)	2019	0.566
Bosnia and Herzegovina	2019	0.718
Botswana	2019	0.634
Brazil	2019	0.64
Brunei Darussalam	2019	0.823
Bulgaria	2019	0.764
Burkina Faso	2019	0.257
Burundi	2019	0.284
Cabo Verde	2019	0.525
Cambodia	2019	0.469
Cameroon	2019	0.49
Canada	2019	0.873
Central African Republic	2019	0.274

Chad	2019	0.238
Chile	2019	0.759
China	2019	0.686
Colombia	2019	0.633
Comoros	2019	0.455
Congo	2019	0.568
Cook Islands	2019	0.764
Costa Rica	2019	0.68
C 么 te d'Ivoire	2019	0.408
Croatia	2019	0.794
Cuba	2019	0.668
Cyprus	2019	0.841
Czechia	2019	0.828
Democratic People's Republic of Korea	2019	0.558
Democratic Republic of the Congo	2019	0.382
Denmark	2019	0.89
Djibouti	2019	0.459
Dominica	2019	0.729
Dominican Republic	2019	0.592
Ecuador	2019	0.64
Egypt	2019	0.658
El Salvador	2019	0.573
Equatorial Guinea	2019	0.685
Eritrea	2019	0.396
Estonia	2019	0.835
Eswatini	2019	0.577
Ethiopia	2019	0.343
Fiji	2019	0.664
Finland	2019	0.856
France	2019	0.834
Gabon	2019	0.656
Gambia	2019	0.399
Georgia	2019	0.702
Germany	2019	0.898
Ghana	2019	0.557
Greece	2019	0.794
Greenland	2019	0.761

Grenada	2019	0.669
Guam	2019	0.813
Guatemala	2019	0.526
Guinea-Bissau	2019	0.355
Guinea	2019	0.325
Guyana	2019	0.618
Haiti	2019	0.432
Honduras	2019	0.496
Hungary	2019	0.791
Iceland	2019	0.869
India	2019	0.566
Indonesia	2019	0.66
Iran (Islamic Republic of)	2019	0.67
Iraq	2019	0.671
Ireland	2019	0.867
Israel	2019	0.803
Italy	2019	0.801
Jamaica	2019	0.684
Japan	2019	0.87
Jordan	2019	0.731
Kazakhstan	2019	0.723
Kenya	2019	0.508
Kiribati	2019	0.527
Kuwait	2019	0.851
Kyrgyzstan	2019	0.596
Lao People's Democratic Republic	2019	0.49
Latvia	2019	0.82
Lebanon	2019	0.708
Lesotho	2019	0.507
Liberia	2019	0.37
Libya	2019	0.709
Lithuania	2019	0.843
Luxembourg	2019	0.895
Madagascar	2019	0.396
Malawi	2019	0.384
Malaysia	2019	0.737
Maldives	2019	0.562
Mali	2019	0.263

Malta	2019	0.801
Marshall Islands	2019	0.544
Mauritania	2019	0.496
Mauritius	2019	0.705
Mexico	2019	0.649
Micronesia (Federated States of)	2019	0.58
Monaco	2019	0.902
Mongolia	2019	0.606
Montenegro	2019	0.791
Morocco	2019	0.548
Mozambique	2019	0.307
Myanmar	2019	0.521
Namibia	2019	0.612
Nauru	2019	0.618
Nepal	2019	0.422
Netherlands	2019	0.883
New Zealand	2019	0.84
Nicaragua	2019	0.517
Niger	2019	0.162
Nigeria	2019	0.515
Niue	2019	0.711
North Macedonia	2019	0.744
Northern Mariana Islands	2019	0.771
Norway	2019	0.913
Oman	2019	0.783
Pakistan	2019	0.449
Palau	2019	0.738
Palestine	2019	0.588
Panama	2019	0.686
Papua New Guinea	2019	0.394
Paraguay	2019	0.638
Peru	2019	0.648
Philippines	2019	0.623
Poland	2019	0.802
Portugal	2019	0.743
Puerto Rico	2019	0.814
Qatar	2019	0.83
Republic of Korea	2019	0.878

Republic of Moldova	2019	0.696
Romania	2019	0.76
Russian Federation	2019	0.805
Rwanda	2019	0.429
Saint Kitts and Nevis	2019	0.746
Saint Lucia	2019	0.67
Saint Vincent and the Grenadines	2019	0.627
Samoa	2019	0.641
San Marino	2019	0.884
Sao Tome and Principe	2019	0.502
Saudi Arabia	2019	0.805
Senegal	2019	0.389
Serbia	2019	0.767
Seychelles	2019	0.724
Sierra Leone	2019	0.347
Singapore	2019	0.861
Slovakia	2019	0.812
Slovenia	2019	0.84
Solomon Islands	2019	0.407
Somalia	2019	0.081
South Africa	2019	0.678
South Sudan	2019	0.363
Spain	2019	0.767
Sri Lanka	2019	0.69
Sudan	2019	0.515
Suriname	2019	0.636
Sweden	2019	0.872
Switzerland	2019	0.929
Syrian Arab Republic	2019	0.619
Tajikistan	2019	0.539
Thailand	2019	0.687
Timor-Leste	2019	0.514
Togo	2019	0.417
Tokelau	2019	0.626
Tonga	2019	0.636
Trinidad and Tobago	2019	0.757
Tunisia	2019	0.672
Turkey	2019	0.748

Turkmenistan	2019	0.67
Tuvalu	2019	0.589
Uganda	2019	0.404
Ukraine	2019	0.736
United Arab Emirates	2019	0.88
United Kingdom	2019	0.847
United Republic of Tanzania	2019	0.423
United States of America	2019	0.859
United States Virgin Islands	2019	0.799
Uruguay	2019	0.697
Uzbekistan	2019	0.631
Vanuatu	2019	0.485
Venezuela (Bolivarian Republic of)	2019	0.607
Viet Nam	2019	0.617
Yemen	2019	0.412
Zambia	2019	0.505
Zimbabwe	2019	0.476

SDI: socio-demographic index

Additional File 3: Supplementary figures

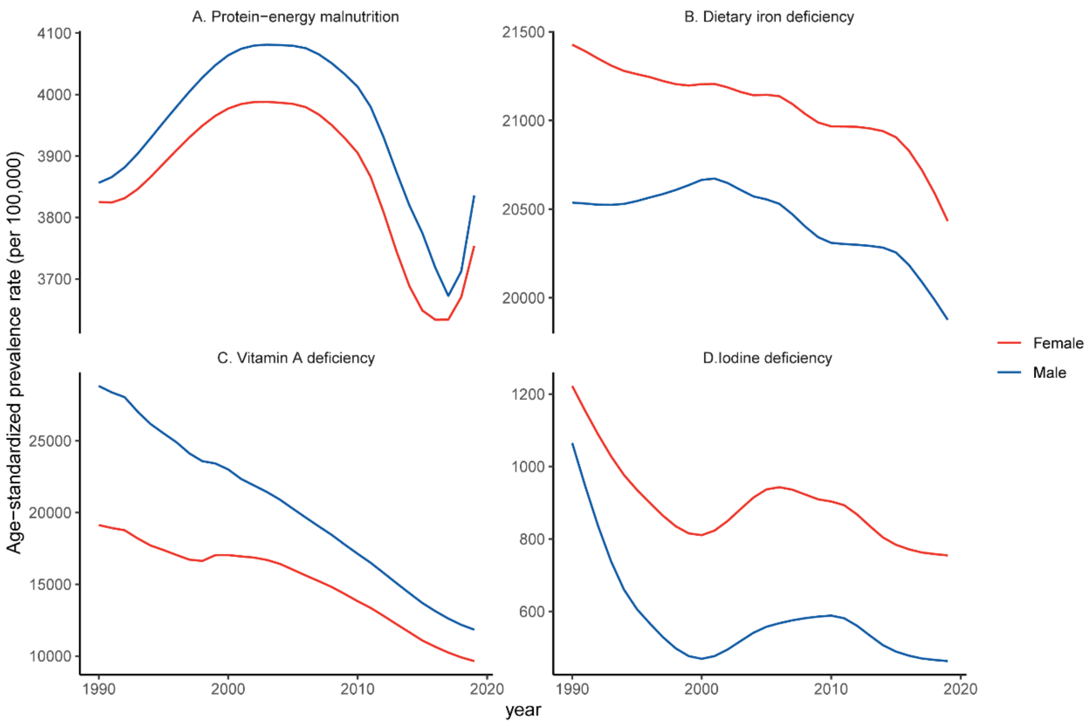

Figure S1 Global age-standardized prevalence rates of protein-energy malnutrition, dietary iron deficiency, vitamin A deficiency and iodine deficiency in males and females, 1990 to 2019

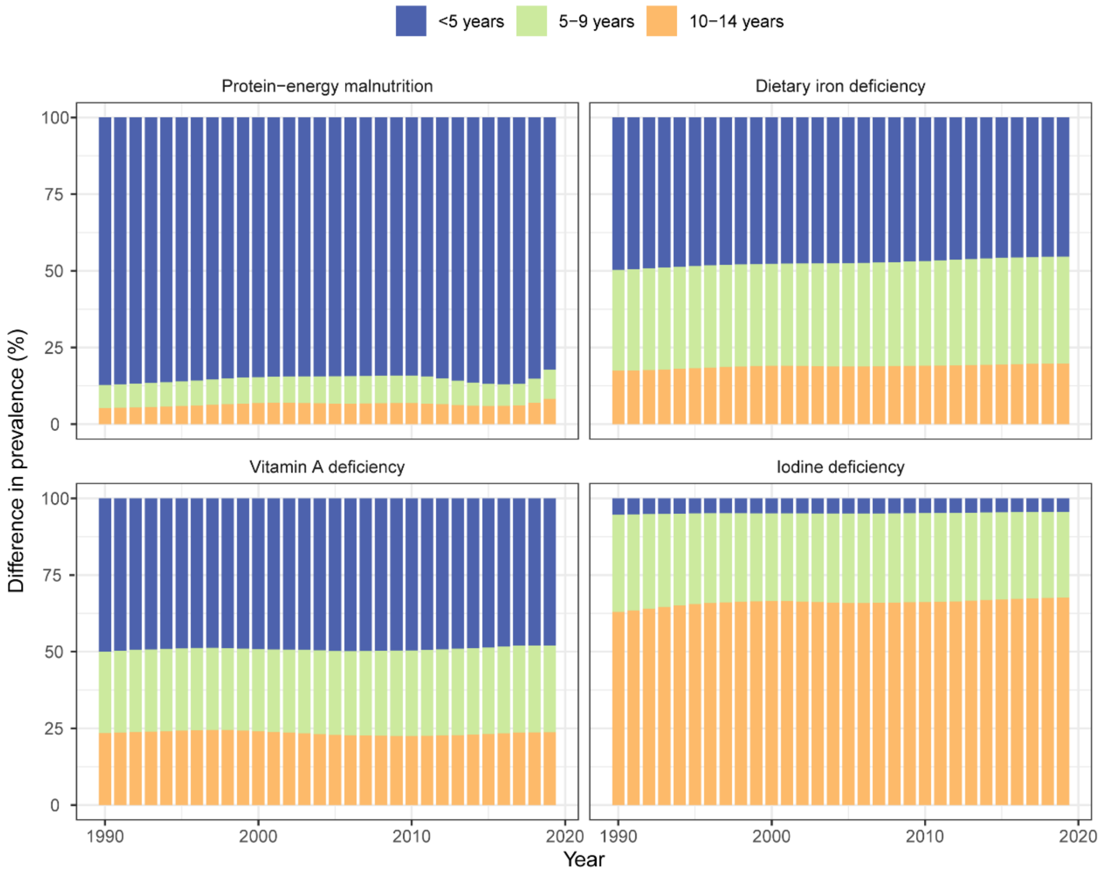

Figure S2 Temporal change in the relative proportion of four nutritional deficiencies prevalence cases across age groups, 1990 to 2019.

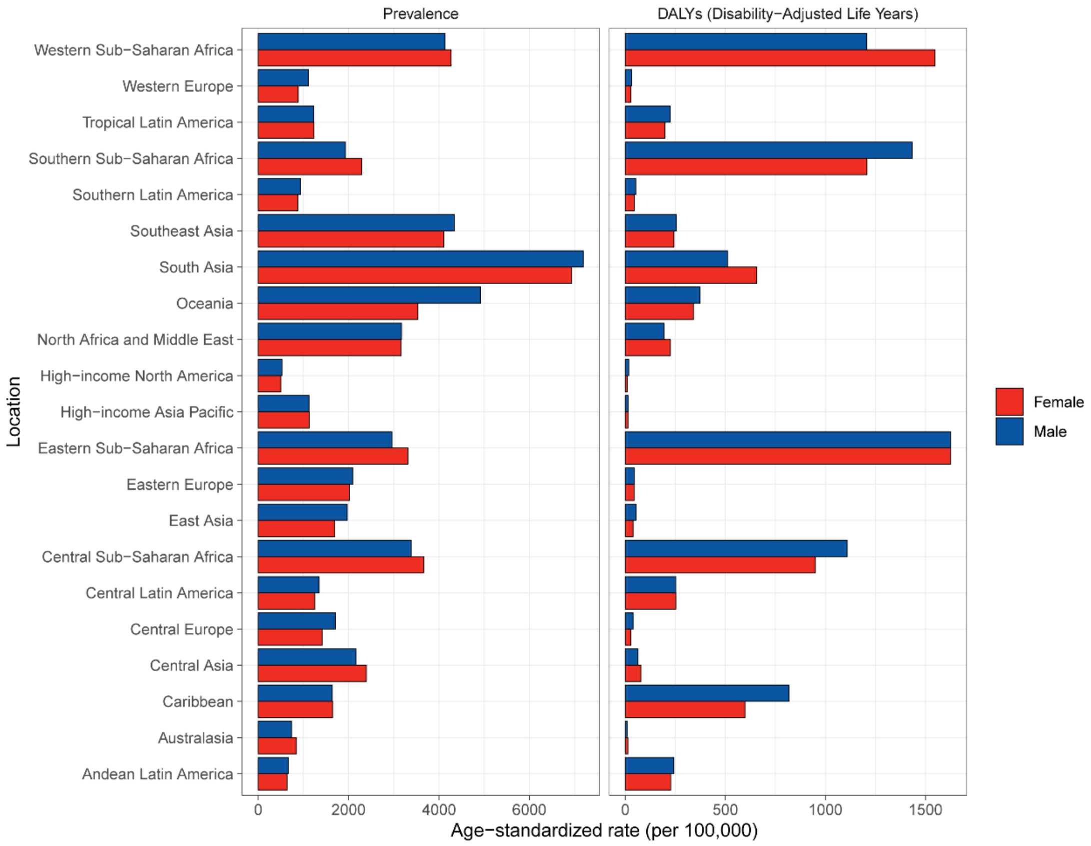

Figure S3 The age-standardized prevalence and DALY rates of protein-energy malnutrition by sex in 21 GBD regions, 2019. DALY: disability-adjusted life year

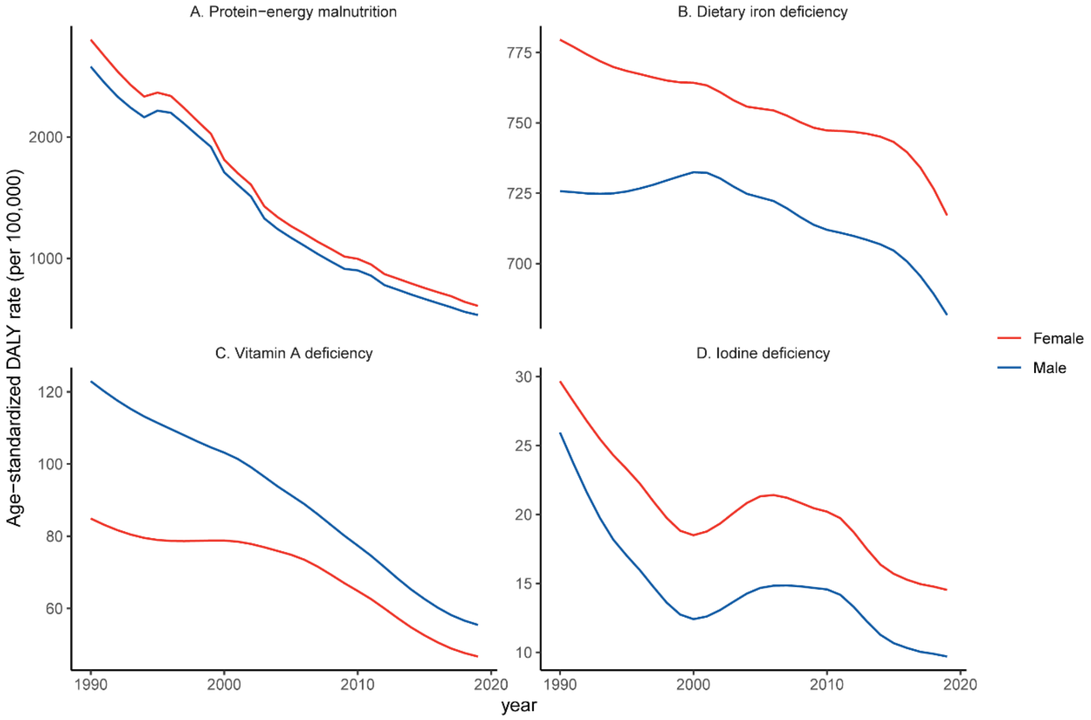

Figure S4 Global age-standardized DALY rates of protein-energy malnutrition, dietary iron deficiency, vitamin A deficiency and iodine deficiency in males and females, 1990 to 2019

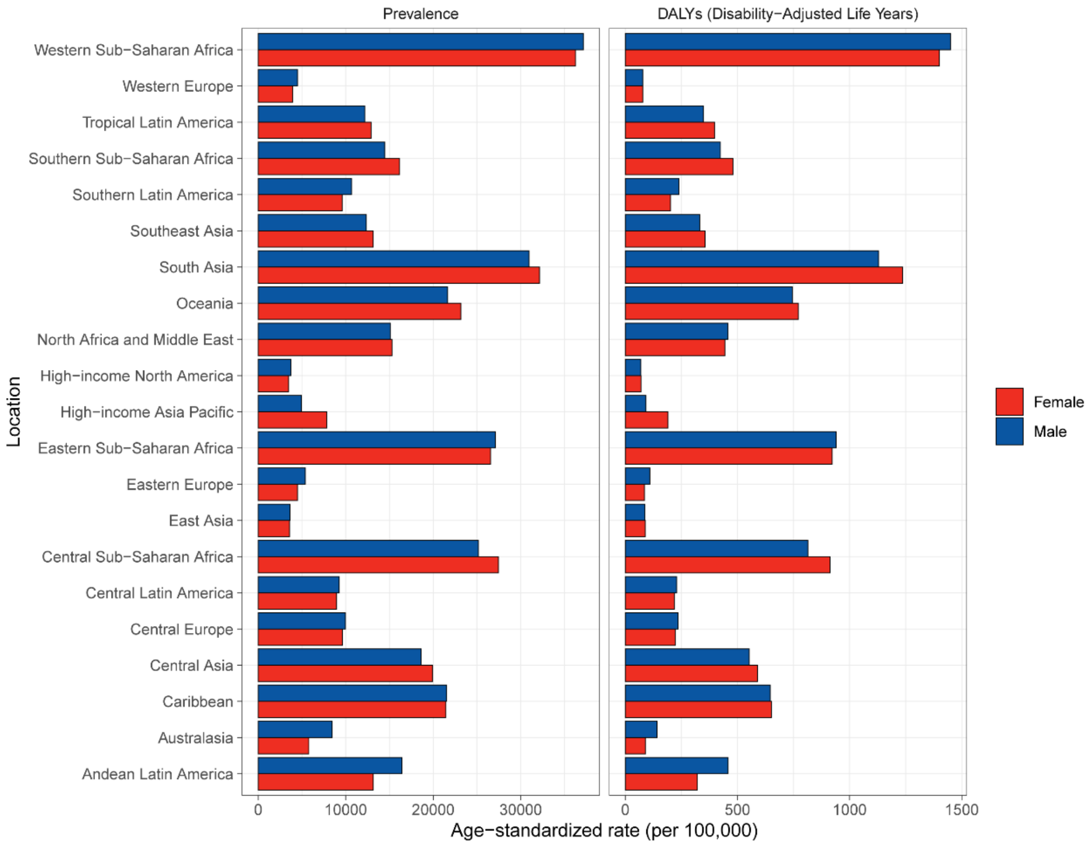

Figure S5 The age-standardized prevalence and DALY rates of dietary iron deficiency by sex in 21 GBD regions, 2019

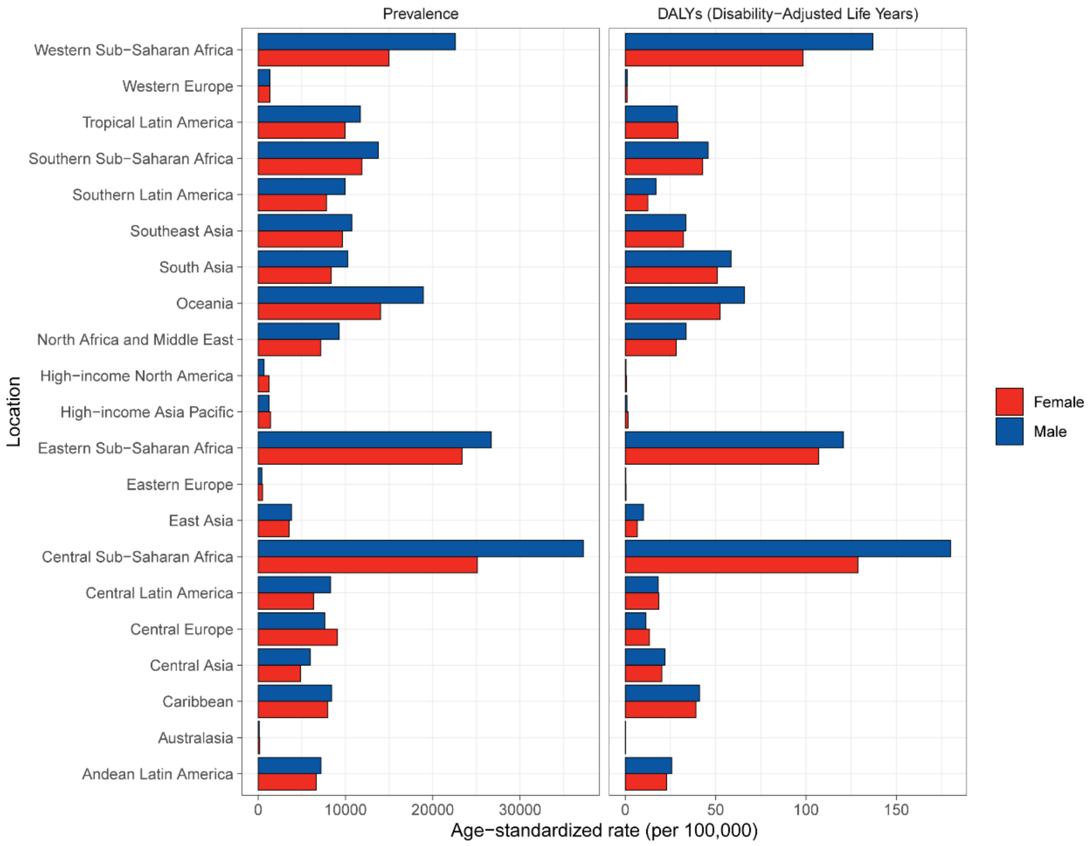

Figure S6 The age-standardized prevalence and DALY rates of vitamin A deficiency by sex in 21 GBD regions, 2019

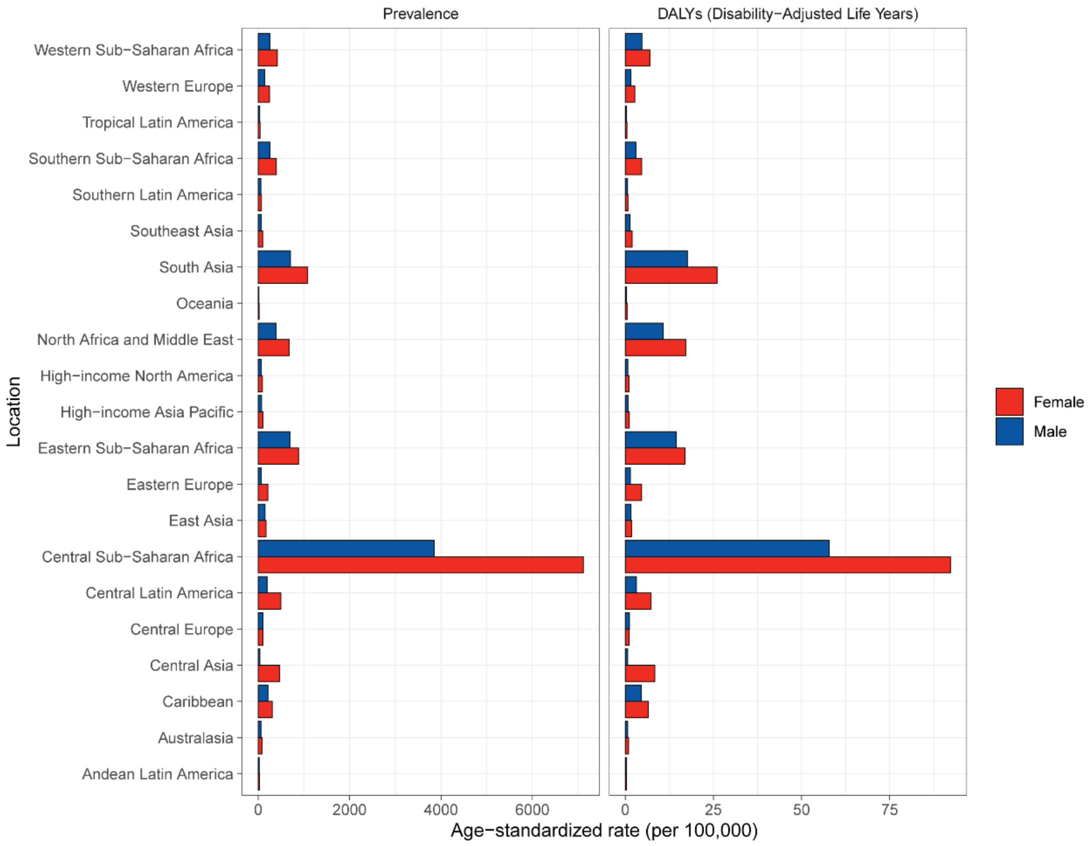

Figure S7 The age-standardized prevalence and DALY rates of iodine deficiency by sex in 21 GBD regions, 2019

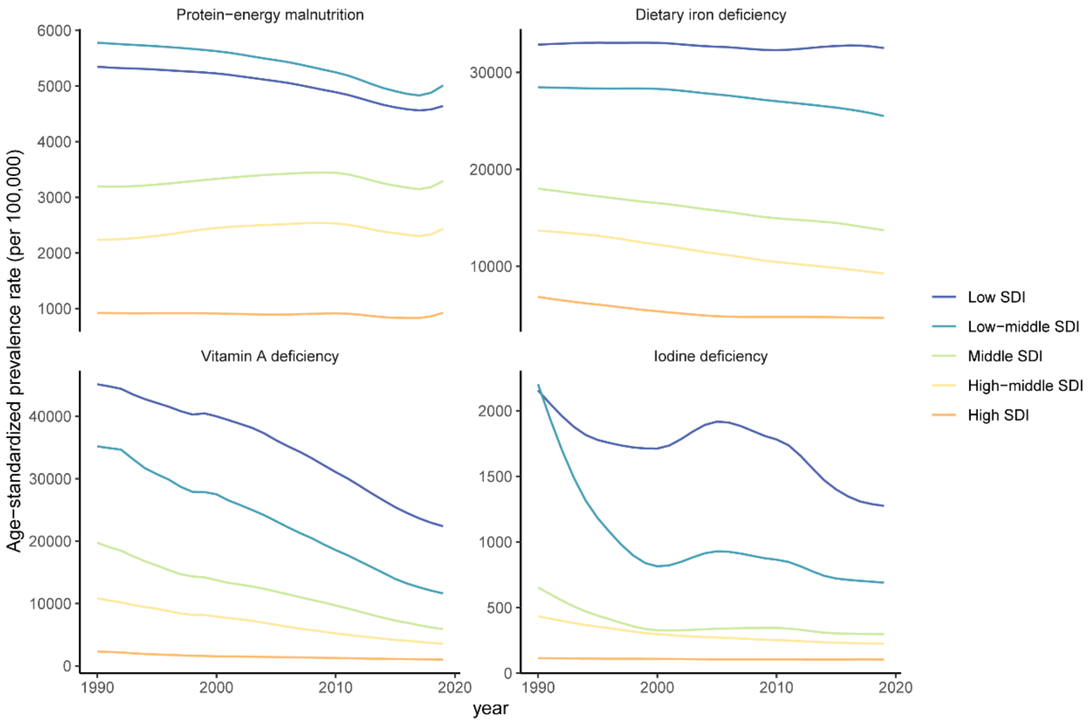

Figure S8 The age-standardized prevalence rates of protein-energy malnutrition, dietary iron deficiency, vitamin A deficiency and iodine deficiency in different SDI quintiles, 1990 to 2019. SDI: socio-demographic index

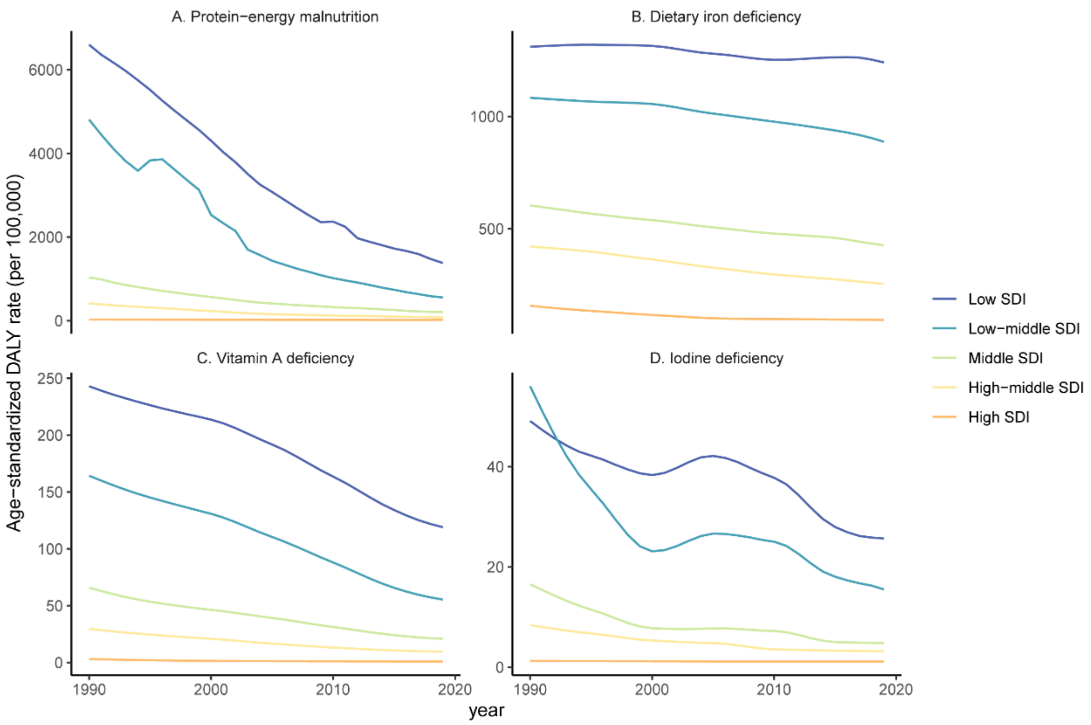

Figure S9 The age-standardized DALY rates of protein-energy malnutrition, dietary iron deficiency, vitamin A deficiency and iodine deficiency in different SDI quintiles, 1990 to 2019. DALY: disability-adjusted life year; SDI: socio-demographic index

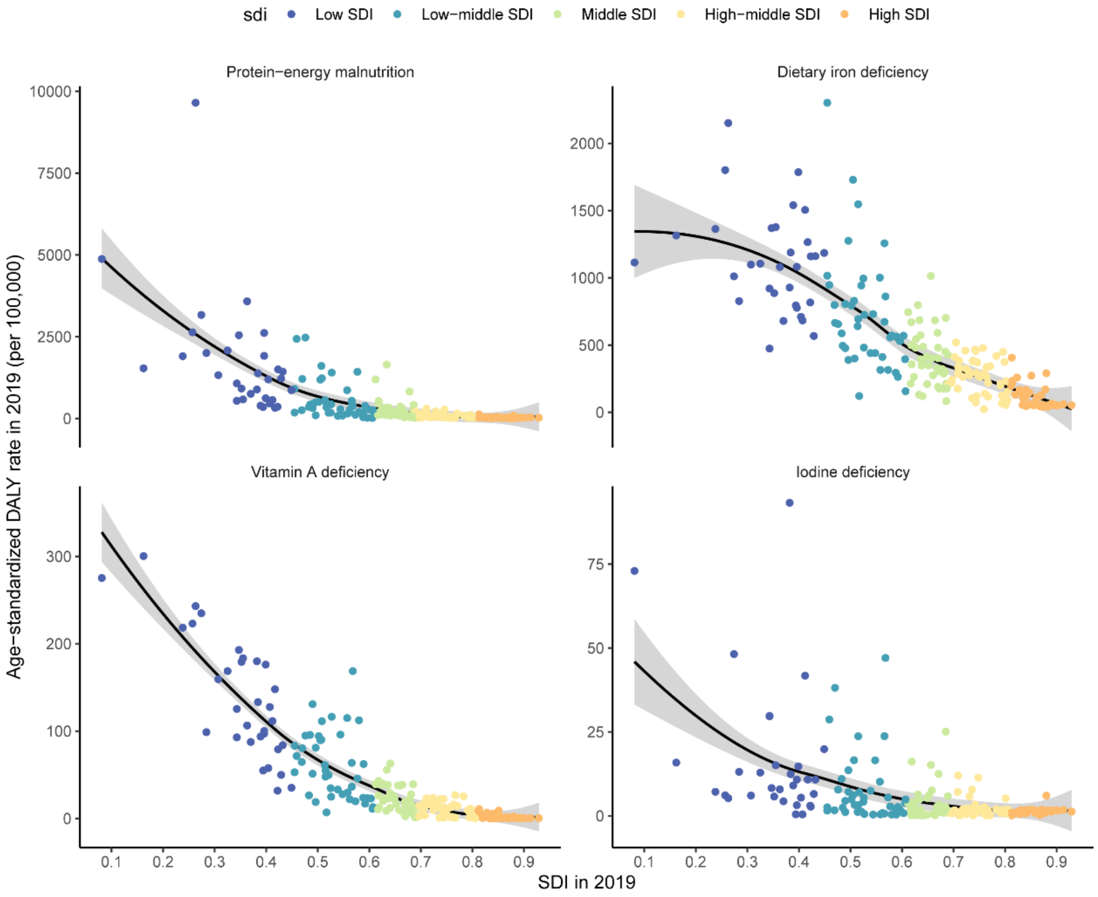

Figure S10 The relationships between age-standardized DALY rates of four nutritional deficiencies and SDI across all countries/territories in 2019. DALY: disability-adjusted life year; SDI: socio-demographic index

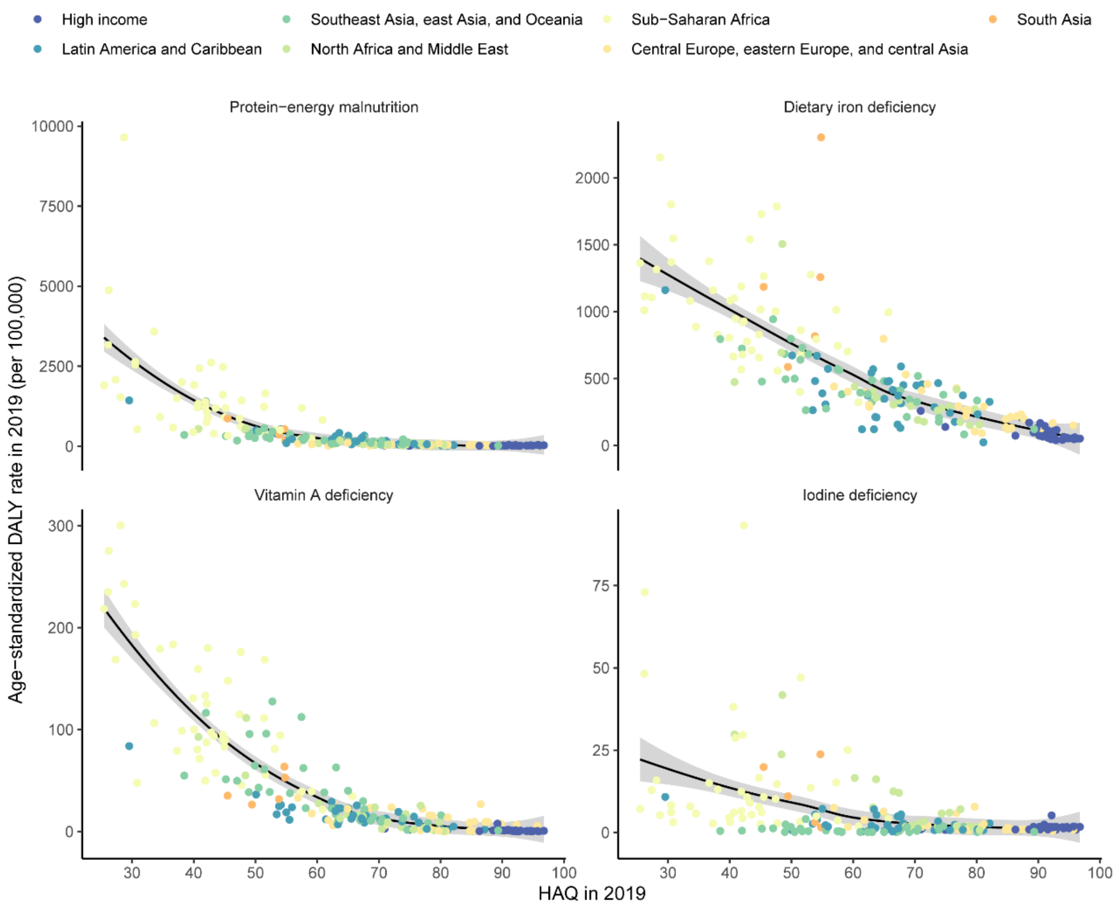

Figure S11 The relationships between age-standardized DALY rates of four nutritional deficiencies and HAQ across all countries/territories in 2019. DALY: disability-adjusted life year; HAQ: Healthcare access and quality

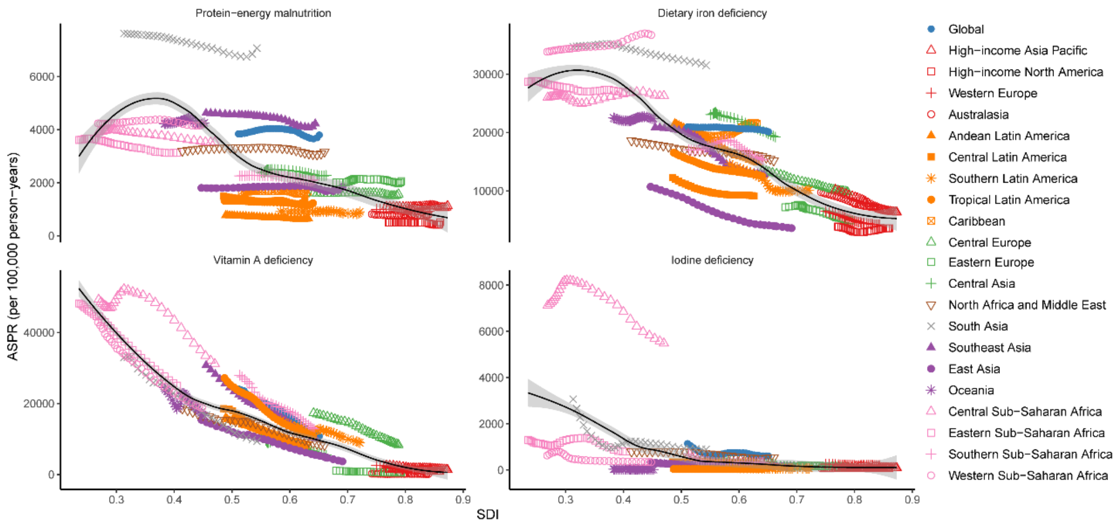

Figure S12 The age-standardized prevalence rates of protein-energy malnutrition, dietary iron deficiency, vitamin A deficiency and iodine deficiency for 21 GBD regions by socio-demographic index, 1990 to 2019. SDI: socio-demographic index; ASPR: age-standardized prevalence rate

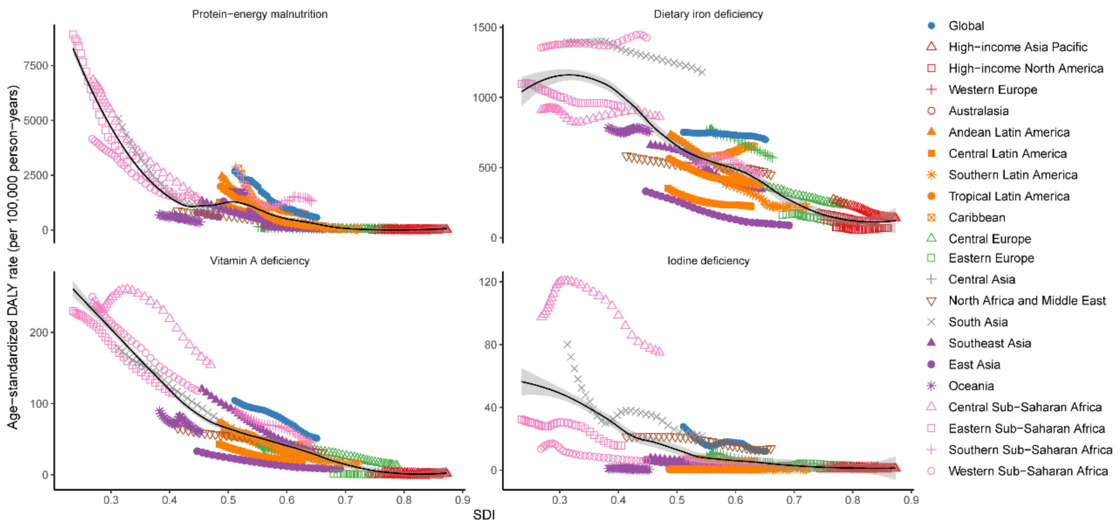

Figure S13 The age-standardized DALY rates of protein-energy malnutrition, dietary iron deficiency, vitamin A deficiency and iodine deficiency for 21 GBD regions by socio-demographic index, 1990 to 2019. SDI: socio-demographic index; DALY: disability-adjusted life year

Additional File 4: Supplementary tables

Table S1 Count and rates of protein-energy malnutrition, dietary iron deficiency, vitamin A deficiency and iodine deficiency prevalence in 21 GBD regions in 2019

location	Cause	Age	Number	Rate
Andean Latin America	Dietary iron deficiency	<5 years	1499751 (1282446-1747029)	23674.4 (20244.11-27577.81)
Andean Latin America	Dietary iron deficiency	10-14 years	308300 (192926-474334)	5406.86 (3383.47-8318.71)
Andean Latin America	Dietary iron deficiency	5-9 years	878669 (618093-1192064)	14514.04 (10209.79-19690.75)
Andean Latin America	Iodine deficiency	<5 years	219 (101-380)	3.46 (1.6-5.99)
Andean Latin America	Iodine deficiency	10-14 years	3038 (1483-5140)	53.28 (26.01-90.15)
Andean Latin America	Iodine deficiency	5-9 years	1377 (635-2378)	22.74 (10.49-39.28)
Andean Latin America	Protein-energy malnutrition	<5 years	98806 (95174-102871)	1559.71 (1502.38-1623.88)
Andean Latin America	Protein-energy malnutrition	10-14 years	10353 (7722-13759)	181.57 (135.42-241.3)
Andean Latin America	Protein-energy malnutrition	5-9 years	9870 (6592-14017)	163.04 (108.89-231.54)
Andean Latin America	Vitamin A deficiency	<5 years	486312 (374576-634286)	7676.71 (5912.89-10012.56)
Andean Latin America	Vitamin A deficiency	10-14 years	369925 (265270-519090)	6487.62 (4652.2-9103.63)
Andean Latin America	Vitamin A deficiency	5-9 years	399772 (282426-561955)	6603.52 (4665.16-9282.48)
Australasia	Dietary iron deficiency	<5 years	280172 (174923-434927)	15400.54 (9615.18-23907.16)
Australasia	Dietary iron deficiency	10-14 years	19023 (6436-45477)	1042.32 (352.63-2491.84)
Australasia	Dietary iron deficiency	5-9 years	79587 (32454-167823)	4327.25 (1764.59-9124.77)
Australasia	Iodine deficiency	<5 years	178 (99-279)	9.79 (5.42-15.31)
Australasia	Iodine deficiency	10-14 years	2767 (1606-4231)	151.62 (88-231.86)
Australasia	Iodine deficiency	5-9 years	1184 (654-1849)	64.37 (35.58-100.55)
Australasia	Protein-energy malnutrition	<5 years	32625 (30954-34366)	1793.33 (1701.51-1889.02)
Australasia	Protein-energy malnutrition	10-14 years	4475 (2914-6745)	245.2 (159.69-369.56)
Australasia	Protein-energy malnutrition	5-9 years	4798 (2984-7446)	260.86 (162.24-404.84)
Australasia	Vitamin A deficiency	<5 years	2742 (1837-4075)	150.71 (100.97-224)

Australasia	Vitamin A deficiency	10-14 years	2650 (1617-4174)	145.18 (88.61-228.71)
Australasia	Vitamin A deficiency	5-9 years	2834 (1754-4349)	154.09 (95.38-236.45)
Caribbean	Dietary iron deficiency	<5 years	1216159 (1096335-1338269)	30787.31 (27753.95-33878.55)
Caribbean	Dietary iron deficiency	10-14 years	442561 (316299-577151)	11614.56 (8300.95-15146.74)
Caribbean	Dietary iron deficiency	5-9 years	829841 (683661-988423)	21152.97 (17426.8-25195.29)
Caribbean	Iodine deficiency	<5 years	1442 (754-2284)	36.5 (19.1-57.83)
Caribbean	Iodine deficiency	10-14 years	20529 (11474-31621)	538.78 (301.13-829.85)
Caribbean	Iodine deficiency	5-9 years	9161 (4786-14430)	233.51 (121.99-367.84)
Caribbean	Protein-energy malnutrition	<5 years	163192 (157426-169041)	4131.24 (3985.26-4279.31)
Caribbean	Protein-energy malnutrition	10-14 years	10818 (7555-15229)	283.91 (198.27-399.68)
Caribbean	Protein-energy malnutrition	5-9 years	13068 (8706-18674)	333.11 (221.92-476.02)
Caribbean	Vitamin A deficiency	<5 years	417030 (322514-544513)	10557.21 (8164.5-13784.44)
Caribbean	Vitamin A deficiency	10-14 years	255642 (174407-375656)	6709.08 (4577.13-9858.72)
Caribbean	Vitamin A deficiency	5-9 years	280669 (188233-403133)	7154.35 (4798.13-10276.02)
Central Asia	Dietary iron deficiency	<5 years	2780802 (2457680-3109680)	29050.18 (25674.63-32485.86)
Central Asia	Dietary iron deficiency	10-14 years	831280 (602398-1105431)	10385.52 (7526-13810.6)
Central Asia	Dietary iron deficiency	5-9 years	1620863 (1251660-2007002)	17385.42 (13425.33-21527.15)
Central Asia	Iodine deficiency	<5 years	3217 (2115-4523)	33.6 (22.09-47.25)
Central Asia	Iodine deficiency	10-14 years	40823 (27726-57244)	510.02 (346.39-715.17)
Central Asia	Iodine deficiency	5-9 years	20513 (13522-28933)	220.02 (145.04-310.33)
Central Asia	Protein-energy malnutrition	<5 years	570770 (551963-589118)	5962.66 (5766.19-6154.34)
Central Asia	Protein-energy malnutrition	10-14 years	20892 (13169-30653)	261.02 (164.52-382.96)
Central Asia	Protein-energy malnutrition	5-9 years	31499 (19775-46327)	337.85 (212.1-496.9)
Central Asia	Vitamin A deficiency	<5 years	684742 (551247-847805)	7153.29 (5758.71-8856.76)
Central Asia	Vitamin A deficiency	10-14 years	311704 (227144-421560)	3894.25 (2837.81-5266.72)
Central Asia	Vitamin A deficiency	5-9 years	473339 (360724-642967)	5077.05 (3869.14-6896.48)

Central Europe	Dietary iron deficiency	<5 years	947281 (797904-1123276)	16759.49 (14116.69-19873.24)
Central Europe	Dietary iron deficiency	10-14 years	240234 (168321-334225)	3959.03 (2773.9-5507.98)
Central Europe	Dietary iron deficiency	5-9 years	476567 (358343-635736)	8055.21 (6056.93-10745.59)
Central Europe	Iodine deficiency	<5 years	801 (500-1172)	14.17 (8.85-20.73)
Central Europe	Iodine deficiency	10-14 years	13340 (8483-19323)	219.85 (139.79-318.43)
Central Europe	Iodine deficiency	5-9 years	5486 (3405-7951)	92.73 (57.55-134.39)
Central Europe	Protein-energy malnutrition	<5 years	191274 (185638-197020)	3384.06 (3284.35-3485.71)
Central Europe	Protein-energy malnutrition	10-14 years	31168 (20770-44111)	513.65 (342.28-726.94)
Central Europe	Protein-energy malnutrition	5-9 years	39193 (26094-54912)	662.47 (441.05-928.15)
Central Europe	Vitamin A deficiency	<5 years	536276 (462364-632274)	9487.91 (8180.24-11186.32)
Central Europe	Vitamin A deficiency	10-14 years	473972 (385189-581613)	7811 (6347.87-9584.91)
Central Europe	Vitamin A deficiency	5-9 years	451617 (367956-547503)	7633.49 (6219.41-9254.21)
Central Latin America	Dietary iron deficiency	<5 years	3400111 (3109805-3731726)	15700.28 (14359.77-17231.54)
Central Latin America	Dietary iron deficiency	10-14 years	706266 (586456-843747)	3205.44 (2661.67-3829.41)
Central Latin America	Dietary iron deficiency	5-9 years	1698486 (1471741-1970686)	7827.64 (6782.66-9082.09)
Central Latin America	Iodine deficiency	<5 years	10030 (6471-14211)	46.31 (29.88-65.62)
Central Latin America	Iodine deficiency	10-14 years	156380 (104782-218603)	709.74 (475.56-992.15)
Central Latin America	Iodine deficiency	5-9 years	66753 (43140-94524)	307.64 (198.82-435.62)
Central Latin America	Protein-energy malnutrition	<5 years	594297 (583887-604904)	2744.21 (2696.15-2793.19)
Central Latin America	Protein-energy malnutrition	10-14 years	116795 (81825-164153)	530.08 (371.37-745.02)
Central Latin America	Protein-energy malnutrition	5-9 years	115423 (76804-167562)	531.94 (353.96-772.23)
Central Latin America	Vitamin A deficiency	<5 years	1916133 (1515626-2465571)	8847.9 (6998.52-11384.97)
Central Latin America	Vitamin A deficiency	10-14 years	1269905 (863182-1828042)	5763.56 (3917.62-8296.71)
Central Latin America	Vitamin A deficiency	5-9 years	1572040 (1078381-2351211)	7244.9 (4969.82-10835.78)
Central Sub-Saharan Africa	Dietary iron deficiency	<5 years	7112666 (6416229-7824470)	34360.08 (30995.71-37798.69)
Central Sub-Saharan Africa	Dietary iron deficiency	10-14 years	2780568 (1972191-3628084)	16402.99 (11634.25-21402.61)

Central Sub-Saharan Africa	Dietary iron deficiency	5-9 years	5286847 (4236786-6455870)	27253.62 (21840.57-33279.92)
Central Sub-Saharan Africa	Iodine deficiency	<5 years	159226 (105111-217015)	769.19 (507.77-1048.36)
Central Sub-Saharan Africa	Iodine deficiency	10-14 years	1908610 (1339575-2500679)	11259.17 (7902.36-14751.88)
Central Sub-Saharan Africa	Iodine deficiency	5-9 years	957834 (644200-1282080)	4937.62 (3320.84-6609.1)
Central Sub-Saharan Africa	Protein-energy malnutrition	<5 years	2005052 (1919074-2094509)	9686.07 (9270.72-10118.22)
Central Sub-Saharan Africa	Protein-energy malnutrition	10-14 years	34611 (25254-46610)	204.17 (148.98-274.96)
Central Sub-Saharan Africa	Protein-energy malnutrition	5-9 years	47796 (33885-65117)	246.39 (174.68-335.68)
Central Sub-Saharan Africa	Vitamin A deficiency	<5 years	9140620 (7581177-10704360)	44156.78 (36623.38-51710.95)
Central Sub-Saharan Africa	Vitamin A deficiency	10-14 years	3891272 (2578583-5338165)	22955.2 (15211.45-31490.63)
Central Sub-Saharan Africa	Vitamin A deficiency	5-9 years	4949044 (3526115-6654513)	25512.25 (18177.07-34303.92)
East Asia	Dietary iron deficiency	<5 years	5263147 (4248108-6384377)	6255.32 (5048.93-7587.91)
East Asia	Dietary iron deficiency	10-14 years	1298665 (846827-1889571)	1771.25 (1154.99-2577.19)
East Asia	Dietary iron deficiency	5-9 years	1993690 (1341577-2807761)	2649.5 (1782.88-3731.35)
East Asia	Iodine deficiency	<5 years	15681 (7949-27336)	18.64 (9.45-32.49)
East Asia	Iodine deficiency	10-14 years	262074 (164199-412811)	357.44 (223.95-563.03)
East Asia	Iodine deficiency	5-9 years	90642 (46086-156757)	120.46 (61.25-208.32)
East Asia	Protein-energy malnutrition	<5 years	2636186 (2480094-2807432)	3133.14 (2947.62-3336.67)
East Asia	Protein-energy malnutrition	10-14 years	805035 (534569-1159610)	1097.99 (729.1-1581.59)
East Asia	Protein-energy malnutrition	5-9 years	910338 (576124-1337035)	1209.79 (765.64-1776.84)
East Asia	Vitamin A deficiency	<5 years	3446249 (2236149-5292442)	4095.91 (2657.69-6290.13)
East Asia	Vitamin A deficiency	10-14 years	2264748 (1343414-3821207)	3088.89 (1832.28-5211.75)
East Asia	Vitamin A deficiency	5-9 years	2868283 (1730043-4624995)	3811.78 (2299.13-6146.35)
Eastern Europe	Dietary iron deficiency	<5 years	1177947 (805960-1695659)	9548.49 (6533.15-13745.09)
Eastern Europe	Dietary iron deficiency	10-14 years	174143 (91987-312684)	1493.33 (788.82-2681.37)
Eastern Europe	Dietary iron deficiency	5-9 years	447608 (232540-816791)	3426.93 (1780.36-6253.45)
Eastern Europe	Iodine deficiency	<5 years	2249 (1498-3169)	18.23 (12.14-25.69)

Eastern Europe	Iodine deficiency	10-14 years	33283 (22847-45920)	285.42 (195.92-393.78)
Eastern Europe	Iodine deficiency	5-9 years	15844 (10575-22302)	121.31 (80.96-170.74)
Eastern Europe	Protein-energy malnutrition	<5 years	602257 (565181-636252)	4881.93 (4581.39-5157.49)
Eastern Europe	Protein-energy malnutrition	10-14 years	53739 (35829-75719)	460.83 (307.25-649.31)
Eastern Europe	Protein-energy malnutrition	5-9 years	82208 (55848-114389)	629.39 (427.58-875.77)
Eastern Europe	Vitamin A deficiency	<5 years	66833 (50228-89429)	541.75 (407.15-724.91)
Eastern Europe	Vitamin A deficiency	10-14 years	46733 (33563-65794)	400.75 (287.82-564.2)
Eastern Europe	Vitamin A deficiency	5-9 years	56009 (38760-79084)	428.81 (296.75-605.48)
Eastern Sub-Saharan Africa	Dietary iron deficiency	<5 years	23196909 (22309235-24069046)	36166.93 (34782.94-37526.71)
Eastern Sub-Saharan Africa	Dietary iron deficiency	10-14 years	8592394 (7615028-9630280)	16037.51 (14213.27-17974.69)
Eastern Sub-Saharan Africa	Dietary iron deficiency	5-9 years	16064781 (14786485-17402971)	27314.29 (25140.86-29589.57)
Eastern Sub-Saharan Africa	Iodine deficiency	<5 years	62786 (36322-99855)	97.89 (56.63-155.69)
Eastern Sub-Saharan Africa	Iodine deficiency	10-14 years	914399 (610881-1326400)	1706.71 (1140.2-2475.69)
Eastern Sub-Saharan Africa	Iodine deficiency	5-9 years	377889 (221110-589610)	642.51 (375.94-1002.49)
Eastern Sub-Saharan Africa	Protein-energy malnutrition	<5 years	5410116 (5336589-5486486)	8435.06 (8320.42-8554.13)
Eastern Sub-Saharan Africa	Protein-energy malnutrition	10-14 years	145797 (108081-192894)	272.13 (201.73-360.03)
Eastern Sub-Saharan Africa	Protein-energy malnutrition	5-9 years	187948 (131873-258016)	319.56 (224.22-438.69)
Eastern Sub-Saharan Africa	Vitamin A deficiency	<5 years	22053625 (19938201-24290274)	34384.41 (31086.19-37871.63)
Eastern Sub-Saharan Africa	Vitamin A deficiency	10-14 years	10258044 (8693240-12053585)	19146.4 (16225.73-22497.74)
Eastern Sub-Saharan Africa	Vitamin A deficiency	5-9 years	12298713 (10460278-14406050)	20911 (17785.19-24494.02)
High-income Asia Pacific	Dietary iron deficiency	<5 years	743010 (515835-1073210)	10197.16 (7079.37-14728.85)
High-income Asia Pacific	Dietary iron deficiency	10-14 years	274887 (139120-500412)	3392.61 (1717-6176)
High-income Asia Pacific	Dietary iron deficiency	5-9 years	408352 (207040-705176)	5134.33 (2603.17-8866.38)
High-income Asia Pacific	Iodine deficiency	<5 years	882 (489-1351)	12.1 (6.71-18.54)
High-income Asia Pacific	Iodine deficiency	10-14 years	15059 (8882-22847)	185.86 (109.62-281.98)
High-income Asia Pacific	Iodine deficiency	5-9 years	6265 (3497-9598)	78.78 (43.97-120.67)

High-income Asia Pacific	Protein-energy malnutrition	<5 years	174013 (171265-176761)	2388.17 (2350.46-2425.89)
High-income Asia Pacific	Protein-energy malnutrition	10-14 years	33811 (22379-48980)	417.29 (276.2-604.5)
High-income Asia Pacific	Protein-energy malnutrition	5-9 years	38233 (24901-55374)	480.72 (313.09-696.23)
High-income Asia Pacific	Vitamin A deficiency	<5 years	111127 (77372-155358)	1525.12 (1061.86-2132.15)
High-income Asia Pacific	Vitamin A deficiency	10-14 years	89650 (57033-135669)	1106.44 (703.89-1674.41)
High-income Asia Pacific	Vitamin A deficiency	5-9 years	105104 (66922-159774)	1321.5 (841.43-2008.88)
High-income North America	Dietary iron deficiency	<5 years	1008124 (641913-1504906)	4804.26 (3059.06-7171.69)
High-income North America	Dietary iron deficiency	10-14 years	478650 (239065-823150)	2037.3 (1017.54-3503.61)
High-income North America	Dietary iron deficiency	5-9 years	843979 (431966-1440954)	3872.79 (1982.17-6612.14)
High-income North America	Iodine deficiency	<5 years	2259 (1246-3545)	10.77 (5.94-16.89)
High-income North America	Iodine deficiency	10-14 years	39088 (22969-59836)	166.37 (97.76-254.68)
High-income North America	Iodine deficiency	5-9 years	15398 (8567-24099)	70.66 (39.31-110.58)
High-income North America	Protein-energy malnutrition	<5 years	105496 (98293-113325)	502.74 (468.42-540.06)
High-income North America	Protein-energy malnutrition	10-14 years	116535 (78594-167685)	496.01 (334.52-713.72)
High-income North America	Protein-energy malnutrition	5-9 years	117715 (75786-173215)	540.16 (347.76-794.84)
High-income North America	Vitamin A deficiency	<5 years	268740 (182664-409632)	1280.69 (870.49-1952.12)
High-income North America	Vitamin A deficiency	10-14 years	151138 (91699-240947)	643.3 (390.3-1025.55)
High-income North America	Vitamin A deficiency	5-9 years	206843 (126176-327033)	949.15 (578.99-1500.67)
North Africa and Middle East	Dietary iron deficiency	<5 years	14484383 (13473126-15480669)	24253.87 (22560.53-25922.13)
North Africa and Middle East	Dietary iron deficiency	10-14 years	3139362 (2464219-3989422)	5546.48 (4353.67-7048.33)
North Africa and Middle East	Dietary iron deficiency	5-9 years	8828320 (7358358-10453357)	14832.05 (12362.44-17562.2)
North Africa and Middle East	Iodine deficiency	<5 years	43956 (29670-59921)	73.6 (49.68-100.34)
North Africa and Middle East	Iodine deficiency	10-14 years	619022 (437820-837949)	1093.66 (773.52-1480.45)
North Africa and Middle East	Iodine deficiency	5-9 years	286025 (196864-389949)	480.54 (330.74-655.14)
North Africa and Middle East	Protein-energy malnutrition	<5 years	4640323 (4561257-4719217)	7770.15 (7637.75-7902.25)
North Africa and Middle East	Protein-energy malnutrition	10-14 years	352621 (243809-496064)	622.99 (430.75-876.42)

North Africa and Middle East	Protein-energy malnutrition	5-9 years	461841 (317355-639044)	775.92 (533.17-1073.63)
North Africa and Middle East	Vitamin A deficiency	<5 years	7546115 (6537272-8548272)	12635.85 (10946.56-14313.94)
North Africa and Middle East	Vitamin A deficiency	10-14 years	2991034 (2359910-3677804)	5284.42 (4169.38-6497.78)
North Africa and Middle East	Vitamin A deficiency	5-9 years	3881312 (3120110-4773375)	6520.81 (5241.95-8019.53)
Oceania	Dietary iron deficiency	<5 years	611333 (523773-704102)	33032.21 (28301.12-38044.82)
Oceania	Dietary iron deficiency	10-14 years	166770 (107919-235896)	11922.11 (7714.97-16863.83)
Oceania	Dietary iron deficiency	5-9 years	333709 (242048-432746)	21115.25 (15315.45-27381.78)
Oceania	Iodine deficiency	<5 years	44 (18-82)	2.38 (0.98-4.41)
Oceania	Iodine deficiency	10-14 years	554 (256-978)	39.62 (18.28-69.89)
Oceania	Iodine deficiency	5-9 years	258 (106-476)	16.31 (6.68-30.11)
Oceania	Protein-energy malnutrition	<5 years	211798 (201820-221723)	11444.13 (10904.95-11980.4)
Oceania	Protein-energy malnutrition	10-14 years	4743 (3504-6342)	339.07 (250.47-453.41)
Oceania	Protein-energy malnutrition	5-9 years	7283 (5120-9884)	460.84 (323.94-625.43)
Oceania	Vitamin A deficiency	<5 years	384257 (285147-511185)	20762.59 (15407.37-27620.95)
Oceania	Vitamin A deficiency	10-14 years	197676 (140976-285429)	14131.58 (10078.17-20404.9)
Oceania	Vitamin A deficiency	5-9 years	229063 (162378-331318)	14493.83 (10274.4-20963.99)
South Asia	Dietary iron deficiency	<5 years	63429223 (60818112-66152032)	38581.2 (36992.98-40237.37)
South Asia	Dietary iron deficiency	10-14 years	38673925 (35561016-42173266)	21522.48 (19790.11-23469.9)
South Asia	Dietary iron deficiency	5-9 years	58002673 (53962437-61820263)	33592.63 (31252.7-35803.61)
South Asia	Iodine deficiency	<5 years	180447 (105053-277263)	109.76 (63.9-168.65)
South Asia	Iodine deficiency	10-14 years	3461473 (2297678-4966393)	1926.35 (1278.68-2763.85)
South Asia	Iodine deficiency	5-9 years	1239888 (718388-1897583)	718.09 (416.06-1099)
South Asia	Protein-energy malnutrition	<5 years	27405626 (27113907-27676881)	16669.64 (16492.2-16834.63)
South Asia	Protein-energy malnutrition	10-14 years	3131087 (2219496-4265128)	1742.49 (1235.17-2373.59)
South Asia	Protein-energy malnutrition	5-9 years	3637885 (2595819-4926060)	2106.9 (1503.39-2852.96)
South Asia	Vitamin A deficiency	<5 years	23244615 (16435167-33202018)	14138.68 (9996.79-20195.33)

South Asia	Vitamin A deficiency	10-14 years	11616282 (6939632-19334080)	6464.59 (3861.98-10759.64)
South Asia	Vitamin A deficiency	5-9 years	12329365 (7604031-19826894)	7140.63 (4403.92-11482.88)
Southeast Asia	Dietary iron deficiency	<5 years	10425945 (9422856-11602684)	19135.53 (17294.49-21295.29)
Southeast Asia	Dietary iron deficiency	10-14 years	3416543 (2565095-4375308)	5939.02 (4458.94-7605.65)
Southeast Asia	Dietary iron deficiency	5-9 years	7082729 (5942479-8370819)	12478.01 (10469.17-14747.31)
Southeast Asia	Iodine deficiency	<5 years	5016 (2425-8841)	9.21 (4.45-16.23)
Southeast Asia	Iodine deficiency	10-14 years	108962 (69733-168628)	189.41 (121.22-293.13)
Southeast Asia	Iodine deficiency	5-9 years	33444 (16055-58532)	58.92 (28.28-103.12)
Southeast Asia	Protein-energy malnutrition	<5 years	5587015 (5502592-5672395)	10254.27 (10099.33-10410.98)
Southeast Asia	Protein-energy malnutrition	10-14 years	553157 (397423-749316)	961.56 (690.85-1302.54)
Southeast Asia	Protein-energy malnutrition	5-9 years	584509 (393256-828155)	1029.76 (692.82-1459)
Southeast Asia	Vitamin A deficiency	<5 years	8485153 (6835710-10367852)	15573.45 (12546.1-19028.91)
Southeast Asia	Vitamin A deficiency	10-14 years	3676103 (2702439-4946269)	6390.22 (4697.68-8598.16)
Southeast Asia	Vitamin A deficiency	5-9 years	4667838 (3460547-6159036)	8223.57 (6096.62-10850.69)
Southern Latin America	Dietary iron deficiency	<5 years	975508 (719513-1255583)	20095.57 (14822.06-25865.16)
Southern Latin America	Dietary iron deficiency	10-14 years	125289 (53248-256918)	2539.74 (1079.39-5208.02)
Southern Latin America	Dietary iron deficiency	5-9 years	356111 (165603-632824)	6957.2 (3235.31-12363.24)
Southern Latin America	Iodine deficiency	<5 years	437 (245-652)	8.99 (5.04-13.44)
Southern Latin America	Iodine deficiency	10-14 years	6698 (3779-9846)	135.78 (76.61-199.58)
Southern Latin America	Iodine deficiency	5-9 years	3013 (1697-4486)	58.87 (33.16-87.64)
Southern Latin America	Protein-energy malnutrition	<5 years	90915 (85773-96060)	1872.85 (1766.94-1978.84)
Southern Latin America	Protein-energy malnutrition	10-14 years	19446 (13068-28122)	394.18 (264.91-570.06)
Southern Latin America	Protein-energy malnutrition	5-9 years	19883 (12248-29504)	388.44 (239.29-576.41)
Southern Latin America	Vitamin A deficiency	<5 years	511617 (360678-710299)	10539.37 (7430-14632.23)
Southern Latin America	Vitamin A deficiency	10-14 years	359108 (233271-552250)	7279.52 (4728.67-11194.73)
Southern Latin America	Vitamin A deficiency	5-9 years	448186 (300300-663071)	8756.03 (5866.85-12954.15)

Southern Sub-Saharan Africa	Dietary iron deficiency	<5 years	1569165 (1371155-1784680)	19381.14 (16935.47-22043.01)
Southern Sub-Saharan Africa	Dietary iron deficiency	10-14 years	762961 (549901-1033084)	10154.27 (7318.66-13749.36)
Southern Sub-Saharan Africa	Dietary iron deficiency	5-9 years	1273562 (979853-1648071)	15911.1 (12241.68-20589.98)
Southern Sub-Saharan Africa	Iodine deficiency	<5 years	3518 (1987-5585)	43.45 (24.54-68.98)
Southern Sub-Saharan Africa	Iodine deficiency	10-14 years	51641 (31711-78999)	687.3 (422.04-1051.41)
Southern Sub-Saharan Africa	Iodine deficiency	5-9 years	22672 (12768-35626)	283.25 (159.52-445.09)
Southern Sub-Saharan Africa	Protein-energy malnutrition	<5 years	439837 (430721-449262)	5432.53 (5319.93-5548.94)
Southern Sub-Saharan Africa	Protein-energy malnutrition	10-14 years	22266 (15857-30329)	296.34 (211.04-403.65)
Southern Sub-Saharan Africa	Protein-energy malnutrition	5-9 years	28548 (19798-40006)	356.66 (247.34-499.81)
Southern Sub-Saharan Africa	Vitamin A deficiency	<5 years	1402683 (1109754-1765146)	17324.88 (13706.84-21801.75)
Southern Sub-Saharan Africa	Vitamin A deficiency	10-14 years	699836 (501256-937513)	9314.14 (6671.24-12477.4)
Southern Sub-Saharan Africa	Vitamin A deficiency	5-9 years	917255 (655958-1236631)	11459.62 (8195.14-15449.71)
Tropical Latin America	Dietary iron deficiency	<5 years	2911740 (2225375-3676599)	18048.8 (13794.27-22789.87)
Tropical Latin America	Dietary iron deficiency	10-14 years	1116417 (561411-1918308)	6576.46 (3307.09-11300.14)
Tropical Latin America	Dietary iron deficiency	5-9 years	2060812 (1247694-3049103)	12411.43 (7514.35-18363.51)
Tropical Latin America	Iodine deficiency	<5 years	813 (404-1365)	5.04 (2.5-8.46)
Tropical Latin America	Iodine deficiency	10-14 years	13152 (7027-21806)	77.48 (41.39-128.45)
Tropical Latin America	Iodine deficiency	5-9 years	5479 (2731-9224)	33 (16.45-55.55)
Tropical Latin America	Protein-energy malnutrition	<5 years	488237 (478843-496526)	3026.4 (2968.17-3077.78)
Tropical Latin America	Protein-energy malnutrition	10-14 years	44143 (29974-63977)	260.03 (176.57-376.87)
Tropical Latin America	Protein-energy malnutrition	5-9 years	45589 (29335-67505)	274.56 (176.67-406.56)
Tropical Latin America	Vitamin A deficiency	<5 years	1873386 (1190131-2866542)	11612.42 (7377.18-17768.63)
Tropical Latin America	Vitamin A deficiency	10-14 years	1748203 (985729-2815801)	10298.1 (5806.61-16586.99)
Tropical Latin America	Vitamin A deficiency	5-9 years	1754845 (990420-2808829)	10568.72 (5964.9-16916.44)
Western Europe	Dietary iron deficiency	<5 years	1589746 (1290584-1941473)	7226.81 (5866.85-8825.72)
Western Europe	Dietary iron deficiency	10-14 years	258338 (162896-388496)	1093.79 (689.7-1644.88)

Western Europe	Dietary iron deficiency	5-9 years	936304 (646391-1350732)	4034.86 (2785.52-5820.78)
Western Europe	Iodine deficiency	<5 years	5289 (2899-8506)	24.04 (13.18-38.67)
Western Europe	Iodine deficiency	10-14 years	98928 (61699-150029)	418.86 (261.23-635.22)
Western Europe	Iodine deficiency	5-9 years	37107 (20470-59105)	159.91 (88.21-254.71)
Western Europe	Protein-energy malnutrition	<5 years	284987 (277828-292396)	1295.52 (1262.98-1329.2)
Western Europe	Protein-energy malnutrition	10-14 years	190685 (131820-268070)	807.35 (558.12-1135)
Western Europe	Protein-energy malnutrition	5-9 years	204931 (135935-290389)	883.12 (585.79-1251.39)
Western Europe	Vitamin A deficiency	<5 years	403925 (339154-486670)	1836.2 (1541.76-2212.35)
Western Europe	Vitamin A deficiency	10-14 years	217363 (172874-272801)	920.31 (731.94-1155.03)
Western Europe	Vitamin A deficiency	5-9 years	293816 (234743-365974)	1266.16 (1011.59-1577.11)
Western Sub-Saharan Africa	Dietary iron deficiency	<5 years	33016023 (31582914-34377114)	45394.17 (43423.77-47265.55)
Western Sub-Saharan Africa	Dietary iron deficiency	10-14 years	13522683 (11067705-16131968)	22773.53 (18639.11-27167.83)
Western Sub-Saharan Africa	Dietary iron deficiency	5-9 years	27019810 (24169979-29526396)	40803.12 (36499.54-44588.36)
Western Sub-Saharan Africa	Iodine deficiency	<5 years	31547 (17794-49893)	43.37 (24.47-68.6)
Western Sub-Saharan Africa	Iodine deficiency	10-14 years	421913 (262075-639865)	710.54 (441.36-1077.6)
Western Sub-Saharan Africa	Iodine deficiency	5-9 years	192176 (108964-301197)	290.21 (164.55-454.84)
Western Sub-Saharan Africa	Protein-energy malnutrition	<5 years	8071685 (7927547-8213444)	11097.87 (10899.69-11292.77)
Western Sub-Saharan Africa	Protein-energy malnutrition	10-14 years	265400 (187336-367413)	446.96 (315.49-618.76)
Western Sub-Saharan Africa	Protein-energy malnutrition	5-9 years	367126 (256284-508695)	554.4 (387.02-768.19)
Western Sub-Saharan Africa	Vitamin A deficiency	<5 years	17529669 (16042554-19333039)	24101.77 (22057.12-26581.25)
Western Sub-Saharan Africa	Vitamin A deficiency	10-14 years	8847157 (7773180-10099117)	14899.49 (13090.8-17007.91)
Western Sub-Saharan Africa	Vitamin A deficiency	5-9 years	11238155 (9908623-12737035)	16970.95 (14963.2-19234.43)

Table S2 Count and rates of protein-energy malnutrition, dietary iron deficiency, vitamin A deficiency and iodine deficiency DALY in 21 GBD regions in 2019

Location	Cause	Age	Number	Rate
Andean Latin America	Dietary iron deficiency	<5 years	32571 (20099-51257)	514.16 (317.28-809.13)
Andean Latin America	Dietary iron deficiency	10-14 years	9896 (5030-18088)	173.55 (88.21-317.22)
Andean Latin America	Dietary iron deficiency	5-9 years	28394 (15432-46625)	469.02 (254.91-770.16)
Andean Latin America	Iodine deficiency	<5 years	3 (1-6)	0.05 (0.02-0.1)
Andean Latin America	Iodine deficiency	10-14 years	41 (16-83)	0.72 (0.28-1.46)
Andean Latin America	Iodine deficiency	5-9 years	19 (7-40)	0.31 (0.11-0.66)
Andean Latin America	Protein-energy malnutrition	<5 years	37911 (25321-52906)	598.45 (399.71-835.15)
Andean Latin America	Protein-energy malnutrition	10-14 years	1719 (1279-2284)	30.14 (22.43-40.06)
Andean Latin America	Protein-energy malnutrition	5-9 years	3028 (2244-3945)	50.01 (37.06-65.16)
Andean Latin America	Vitamin A deficiency	<5 years	2010 (1259-3123)	31.73 (19.87-49.29)
Andean Latin America	Vitamin A deficiency	10-14 years	766 (427-1251)	13.43 (7.49-21.94)
Andean Latin America	Vitamin A deficiency	5-9 years	1625 (936-2604)	26.84 (15.47-43.01)
Australasia	Dietary iron deficiency	<5 years	3880 (1741-7776)	213.3 (95.68-427.43)
Australasia	Dietary iron deficiency	10-14 years	447 (135-1096)	24.49 (7.41-60.03)
Australasia	Dietary iron deficiency	5-9 years	1868 (634-4190)	101.56 (34.48-227.82)
Australasia	Iodine deficiency	<5 years	2 (1-5)	0.11 (0.03-0.25)
Australasia	Iodine deficiency	10-14 years	30 (12-63)	1.64 (0.64-3.46)
Australasia	Iodine deficiency	5-9 years	13 (5-27)	0.7 (0.25-1.45)
Australasia	Protein-energy malnutrition	<5 years	208 (138-304)	11.44 (7.57-16.7)
Australasia	Protein-energy malnutrition	10-14 years	195 (99-343)	10.66 (5.44-18.81)
Australasia	Protein-energy malnutrition	5-9 years	208 (97-380)	11.33 (5.29-20.64)
Australasia	Vitamin A deficiency	<5 years	6 (3-11)	0.31 (0.14-0.62)
Australasia	Vitamin A deficiency	10-14 years	1 (0-2)	0.04 (0.01-0.11)
Australasia	Vitamin A deficiency	5-9 years	3 (1-8)	0.18 (0.05-0.45)

Caribbean	Dietary iron deficiency	<5 years	31271 (19929-46914)	791.62 (504.5-1187.64)
Caribbean	Dietary iron deficiency	10-14 years	15130 (8381-25104)	397.06 (219.96-658.84)
Caribbean	Dietary iron deficiency	5-9 years	28895 (17041-44899)	736.55 (434.38-1144.49)
Caribbean	Iodine deficiency	<5 years	31 (13-60)	0.79 (0.32-1.51)
Caribbean	Iodine deficiency	10-14 years	427 (193-780)	11.2 (5.06-20.47)
Caribbean	Iodine deficiency	5-9 years	201 (80-394)	5.12 (2.04-10.04)
Caribbean	Protein-energy malnutrition	<5 years	73942 (46640-114176)	1871.86 (1180.69-2890.39)
Caribbean	Protein-energy malnutrition	10-14 years	1847 (1304-2615)	48.49 (34.23-68.64)
Caribbean	Protein-energy malnutrition	5-9 years	4849 (3252-7055)	123.6 (82.88-179.83)
Caribbean	Vitamin A deficiency	<5 years	2213 (1356-3425)	56.03 (34.33-86.7)
Caribbean	Vitamin A deficiency	10-14 years	881 (466-1479)	23.12 (12.22-38.82)
Caribbean	Vitamin A deficiency	5-9 years	1548 (839-2581)	39.47 (21.39-65.78)
Central Asia	Dietary iron deficiency	<5 years	70737 (43041-106458)	738.97 (449.64-1112.14)
Central Asia	Dietary iron deficiency	10-14 years	28243 (15610-45041)	352.85 (195.02-562.72)
Central Asia	Dietary iron deficiency	5-9 years	55820 (33860-86710)	598.72 (363.18-930.06)
Central Asia	Iodine deficiency	<5 years	60 (31-105)	0.63 (0.32-1.1)
Central Asia	Iodine deficiency	10-14 years	718 (360-1272)	8.97 (4.5-15.89)
Central Asia	Iodine deficiency	5-9 years	376 (169-664)	4.03 (1.81-7.12)
Central Asia	Protein-energy malnutrition	<5 years	16979 (11646-23389)	177.38 (121.67-244.34)
Central Asia	Protein-energy malnutrition	10-14 years	949 (544-1546)	11.86 (6.79-19.31)
Central Asia	Protein-energy malnutrition	5-9 years	1396 (783-2285)	14.98 (8.4-24.51)
Central Asia	Vitamin A deficiency	<5 years	2922 (1842-4399)	30.53 (19.24-45.96)
Central Asia	Vitamin A deficiency	10-14 years	872 (505-1379)	10.9 (6.3-17.23)
Central Asia	Vitamin A deficiency	5-9 years	1937 (1185-3027)	20.78 (12.71-32.46)
Central Europe	Dietary iron deficiency	<5 years	18108 (10851-28372)	320.37 (191.98-501.96)
Central Europe	Dietary iron deficiency	10-14 years	7205 (4064-12019)	118.74 (66.98-198.07)

Central Europe	Dietary iron deficiency	5-9 years	14173 (7931-23080)	239.56 (134.05-390.12)
Central Europe	Iodine deficiency	<5 years	9 (3-17)	0.15 (0.06-0.3)
Central Europe	Iodine deficiency	10-14 years	146 (59-294)	2.4 (0.97-4.85)
Central Europe	Iodine deficiency	5-9 years	60 (24-125)	1.02 (0.41-2.11)
Central Europe	Protein-energy malnutrition	<5 years	3036 (2139-4147)	53.72 (37.84-73.37)
Central Europe	Protein-energy malnutrition	10-14 years	1179 (668-1885)	19.42 (11-31.06)
Central Europe	Protein-energy malnutrition	5-9 years	1483 (847-2373)	25.07 (14.32-40.11)
Central Europe	Vitamin A deficiency	<5 years	1070 (638-1679)	18.93 (11.28-29.7)
Central Europe	Vitamin A deficiency	10-14 years	361 (201-612)	5.94 (3.31-10.08)
Central Europe	Vitamin A deficiency	5-9 years	692 (387-1139)	11.7 (6.53-19.26)
Central Latin America	Dietary iron deficiency	<5 years	67981 (43507-101307)	313.91 (200.9-467.79)
Central Latin America	Dietary iron deficiency	10-14 years	22506 (14039-35170)	102.15 (63.72-159.62)
Central Latin America	Dietary iron deficiency	5-9 years	53289 (33806-79175)	245.59 (155.8-364.89)
Central Latin America	Iodine deficiency	<5 years	160 (74-291)	0.74 (0.34-1.35)
Central Latin America	Iodine deficiency	10-14 years	2308 (1123-4186)	10.47 (5.1-19)
Central Latin America	Iodine deficiency	5-9 years	1068 (517-1950)	4.92 (2.38-8.99)
Central Latin America	Protein-energy malnutrition	<5 years	135494 (99480-179783)	625.66 (459.36-830.16)
Central Latin America	Protein-energy malnutrition	10-14 years	9834 (8070-12058)	44.63 (36.63-54.73)
Central Latin America	Protein-energy malnutrition	5-9 years	13214 (10944-15911)	60.9 (50.44-73.33)
Central Latin America	Vitamin A deficiency	<5 years	5559 (3515-8280)	25.67 (16.23-38.24)
Central Latin America	Vitamin A deficiency	10-14 years	2120 (1221-3432)	9.62 (5.54-15.58)
Central Latin America	Vitamin A deficiency	5-9 years	4101 (2529-6246)	18.9 (11.66-28.79)
Central Sub-Saharan Africa	Dietary iron deficiency	<5 years	194340 (122226-288428)	938.83 (590.45-1393.35)
Central Sub-Saharan Africa	Dietary iron deficiency	10-14 years	102117 (55670-164033)	602.41 (328.4-967.65)
Central Sub-Saharan Africa	Dietary iron deficiency	5-9 years	199659 (119634-306269)	1029.24 (616.71-1578.81)
Central Sub-Saharan Africa	Iodine deficiency	<5 years	3260 (1547-5568)	15.75 (7.48-26.9)

Central Sub-Saharan Africa	Iodine deficiency	10-14 years	24117 (12009-44952)	142.27 (70.85-265.18)
Central Sub-Saharan Africa	Iodine deficiency	5-9 years	14243 (7560-25363)	73.42 (38.97-130.74)
Central Sub-Saharan Africa	Protein-energy malnutrition	<5 years	571118 (378023-837736)	2758.97 (1826.16-4046.96)
Central Sub-Saharan Africa	Protein-energy malnutrition	10-14 years	11272 (7477-16828)	66.49 (44.11-99.27)
Central Sub-Saharan Africa	Protein-energy malnutrition	5-9 years	27185 (18051-40164)	140.14 (93.05-207.04)
Central Sub-Saharan Africa	Vitamin A deficiency	<5 years	46660 (29882-68609)	225.41 (144.35-331.44)
Central Sub-Saharan Africa	Vitamin A deficiency	10-14 years	15043 (8511-24164)	88.74 (50.21-142.55)
Central Sub-Saharan Africa	Vitamin A deficiency	5-9 years	27663 (16024-43301)	142.6 (82.6-223.22)
East Asia	Dietary iron deficiency	<5 years	105723 (59605-167665)	125.65 (70.84-199.27)
East Asia	Dietary iron deficiency	10-14 years	40891 (21472-71156)	55.77 (29.29-97.05)
East Asia	Dietary iron deficiency	5-9 years	59520 (30529-101227)	79.1 (40.57-134.52)
East Asia	Iodine deficiency	<5 years	171 (63-375)	0.2 (0.07-0.45)
East Asia	Iodine deficiency	10-14 years	2873 (1181-5859)	3.92 (1.61-7.99)
East Asia	Iodine deficiency	5-9 years	995 (368-2190)	1.32 (0.49-2.91)
East Asia	Protein-energy malnutrition	<5 years	62764 (50332-76097)	74.6 (59.82-90.44)
East Asia	Protein-energy malnutrition	10-14 years	21990 (11995-36740)	29.99 (16.36-50.11)
East Asia	Protein-energy malnutrition	5-9 years	26014 (14526-43393)	34.57 (19.3-57.67)
East Asia	Vitamin A deficiency	<5 years	10606 (6639-15854)	12.61 (7.89-18.84)
East Asia	Vitamin A deficiency	10-14 years	3436 (1914-5649)	4.69 (2.61-7.7)
East Asia	Vitamin A deficiency	5-9 years	5926 (3456-9363)	7.88 (4.59-12.44)
Eastern Europe	Dietary iron deficiency	<5 years	18391 (9451-33192)	149.08 (76.61-269.06)
Eastern Europe	Dietary iron deficiency	10-14 years	5124 (2334-10014)	43.94 (20.01-85.88)
Eastern Europe	Dietary iron deficiency	5-9 years	12431 (5465-24340)	95.17 (41.84-186.35)
Eastern Europe	Iodine deficiency	<5 years	50 (24-86)	0.41 (0.19-0.69)
Eastern Europe	Iodine deficiency	10-14 years	713 (359-1227)	6.12 (3.08-10.52)
Eastern Europe	Iodine deficiency	5-9 years	356 (175-600)	2.72 (1.34-4.59)

Eastern Europe	Protein-energy malnutrition	<5 years	9567 (6501-13462)	77.55 (52.7-109.12)
Eastern Europe	Protein-energy malnutrition	10-14 years	2646 (1479-4256)	22.69 (12.69-36.5)
Eastern Europe	Protein-energy malnutrition	5-9 years	4032 (2277-6514)	30.87 (17.43-49.87)
Eastern Europe	Vitamin A deficiency	<5 years	77 (40-139)	0.62 (0.32-1.13)
Eastern Europe	Vitamin A deficiency	10-14 years	16 (7-32)	0.14 (0.06-0.28)
Eastern Europe	Vitamin A deficiency	5-9 years	41 (17-87)	0.32 (0.13-0.67)
Eastern Sub-Saharan Africa	Dietary iron deficiency	<5 years	700470 (464286-1018589)	1092.12 (723.88-1588.11)
Eastern Sub-Saharan Africa	Dietary iron deficiency	10-14 years	330175 (217733-485352)	616.26 (406.39-905.9)
Eastern Sub-Saharan Africa	Dietary iron deficiency	5-9 years	623103 (408575-922291)	1059.44 (694.68-1568.13)
Eastern Sub-Saharan Africa	Iodine deficiency	<5 years	1549 (757-2778)	2.42 (1.18-4.33)
Eastern Sub-Saharan Africa	Iodine deficiency	10-14 years	16756 (9180-27854)	31.27 (17.13-51.99)
Eastern Sub-Saharan Africa	Iodine deficiency	5-9 years	8750 (4390-15184)	14.88 (7.46-25.82)
Eastern Sub-Saharan Africa	Protein-energy malnutrition	<5 years	2750984 (2088024-3602398)	4289.13 (3255.5-5616.6)
Eastern Sub-Saharan Africa	Protein-energy malnutrition	10-14 years	66621 (51856-82613)	124.35 (96.79-154.19)
Eastern Sub-Saharan Africa	Protein-energy malnutrition	5-9 years	157377 (121832-201603)	267.58 (207.15-342.78)
Eastern Sub-Saharan Africa	Vitamin A deficiency	<5 years	108236 (72084-155433)	168.75 (112.39-242.34)
Eastern Sub-Saharan Africa	Vitamin A deficiency	10-14 years	33751 (21963-49710)	63 (40.99-92.78)
Eastern Sub-Saharan Africa	Vitamin A deficiency	5-9 years	61727 (40646-89664)	104.95 (69.11-152.45)
High-income Asia Pacific	Dietary iron deficiency	<5 years	13026 (7063-22206)	178.76 (96.94-304.76)
High-income Asia Pacific	Dietary iron deficiency	10-14 years	7702 (3154-15237)	95.06 (38.93-188.06)
High-income Asia Pacific	Dietary iron deficiency	5-9 years	11202 (4689-21947)	140.85 (58.96-275.95)
High-income Asia Pacific	Iodine deficiency	<5 years	10 (4-21)	0.13 (0.05-0.28)
High-income Asia Pacific	Iodine deficiency	10-14 years	164 (63-342)	2.03 (0.78-4.23)
High-income Asia Pacific	Iodine deficiency	5-9 years	68 (26-142)	0.86 (0.33-1.78)
High-income Asia Pacific	Protein-energy malnutrition	<5 years	1087 (759-1462)	14.91 (10.41-20.06)
High-income Asia Pacific	Protein-energy malnutrition	10-14 years	1019 (560-1627)	12.57 (6.91-20.09)

High-income Asia Pacific	Protein-energy malnutrition	5-9 years	1150 (635-1919)	14.46 (7.99-24.13)
High-income Asia Pacific	Vitamin A deficiency	<5 years	124 (62-234)	1.7 (0.84-3.21)
High-income Asia Pacific	Vitamin A deficiency	10-14 years	57 (21-126)	0.7 (0.25-1.56)
High-income Asia Pacific	Vitamin A deficiency	5-9 years	100 (37-221)	1.25 (0.47-2.77)
High-income North America	Dietary iron deficiency	<5 years	12402 (5859-23250)	59.1 (27.92-110.8)
High-income North America	Dietary iron deficiency	10-14 years	12978 (5391-25210)	55.24 (22.95-107.3)
High-income North America	Dietary iron deficiency	5-9 years	20328 (8488-39682)	93.28 (38.95-182.09)
High-income North America	Iodine deficiency	<5 years	25 (9-53)	0.12 (0.04-0.25)
High-income North America	Iodine deficiency	10-14 years	426 (169-895)	1.81 (0.72-3.81)
High-income North America	Iodine deficiency	5-9 years	169 (66-349)	0.77 (0.3-1.6)
High-income North America	Protein-energy malnutrition	<5 years	1010 (911-1114)	4.81 (4.34-5.31)
High-income North America	Protein-energy malnutrition	10-14 years	4149 (2242-6891)	17.66 (9.54-29.33)
High-income North America	Protein-energy malnutrition	5-9 years	4220 (2249-7013)	19.36 (10.32-32.18)
High-income North America	Vitamin A deficiency	<5 years	126 (55-252)	0.6 (0.26-1.2)
High-income North America	Vitamin A deficiency	10-14 years	59 (19-137)	0.25 (0.08-0.58)
High-income North America	Vitamin A deficiency	5-9 years	152 (55-336)	0.7 (0.25-1.54)
North Africa and Middle East	Dietary iron deficiency	<5 years	398060 (256392-587535)	666.55 (429.32-983.82)
North Africa and Middle East	Dietary iron deficiency	10-14 years	97533 (58676-149969)	172.32 (103.67-264.96)
North Africa and Middle East	Dietary iron deficiency	5-9 years	290779 (178465-457909)	488.52 (299.83-769.31)
North Africa and Middle East	Iodine deficiency	<5 years	1244 (693-2054)	2.08 (1.16-3.44)
North Africa and Middle East	Iodine deficiency	10-14 years	15545 (9022-24982)	27.46 (15.94-44.14)
North Africa and Middle East	Iodine deficiency	5-9 years	7892 (4484-12837)	13.26 (7.53-21.57)
North Africa and Middle East	Protein-energy malnutrition	<5 years	313337 (231803-440201)	524.68 (388.15-737.11)
North Africa and Middle East	Protein-energy malnutrition	10-14 years	19703 (13970-27187)	34.81 (24.68-48.03)
North Africa and Middle East	Protein-energy malnutrition	5-9 years	26714 (19270-36554)	44.88 (32.37-61.41)
North Africa and Middle East	Vitamin A deficiency	<5 years	32313 (21320-46582)	54.11 (35.7-78)

North Africa and Middle East	Vitamin A deficiency	10-14 years	6249 (3670-9564)	11.04 (6.48-16.9)
North Africa and Middle East	Vitamin A deficiency	5-9 years	15340 (9587-23144)	25.77 (16.11-38.88)
Oceania	Dietary iron deficiency	<5 years	18012 (11156-27242)	973.26 (602.8-1471.95)
Oceania	Dietary iron deficiency	10-14 years	6318 (3184-11026)	451.67 (227.64-788.23)
Oceania	Dietary iron deficiency	5-9 years	12946 (7498-20598)	819.16 (474.42-1303.33)
Oceania	Iodine deficiency	<5 years	1 (0-2)	0.05 (0.02-0.11)
Oceania	Iodine deficiency	10-14 years	14 (5-28)	0.98 (0.35-2.01)
Oceania	Iodine deficiency	5-9 years	6 (2-14)	0.41 (0.13-0.9)
Oceania	Protein-energy malnutrition	<5 years	17517 (12765-24479)	946.5 (689.73-1322.68)
Oceania	Protein-energy malnutrition	10-14 years	502 (338-790)	35.87 (24.16-56.45)
Oceania	Protein-energy malnutrition	5-9 years	758 (511-1096)	47.95 (32.32-69.32)
Oceania	Vitamin A deficiency	<5 years	1660 (969-2637)	89.72 (52.35-142.5)
Oceania	Vitamin A deficiency	10-14 years	430 (227-711)	30.71 (16.21-50.85)
Oceania	Vitamin A deficiency	5-9 years	869 (483-1445)	54.98 (30.57-91.42)
South Asia	Dietary iron deficiency	<5 years	1969363 (1313599-2945059)	1197.88 (799-1791.35)
South Asia	Dietary iron deficiency	10-14 years	1613308 (1040745-2324215)	897.82 (579.19-1293.45)
South Asia	Dietary iron deficiency	5-9 years	2463506 (1616120-3596874)	1426.76 (935.99-2083.15)
South Asia	Iodine deficiency	<5 years	4759 (2477-8081)	2.89 (1.51-4.92)
South Asia	Iodine deficiency	10-14 years	82587 (47731-131764)	45.96 (26.56-73.33)
South Asia	Iodine deficiency	5-9 years	31636 (16609-53806)	18.32 (9.62-31.16)
South Asia	Protein-energy malnutrition	<5 years	2473534 (1949284-3170121)	1504.54 (1185.66-1928.25)
South Asia	Protein-energy malnutrition	10-14 years	114373 (71788-169023)	63.65 (39.95-94.06)
South Asia	Protein-energy malnutrition	5-9 years	187252 (126929-257925)	108.45 (73.51-149.38)
South Asia	Vitamin A deficiency	<5 years	138030 (83727-211865)	83.96 (50.93-128.87)
South Asia	Vitamin A deficiency	10-14 years	55134 (31055-88317)	30.68 (17.28-49.15)
South Asia	Vitamin A deficiency	5-9 years	81972 (47607-135165)	47.47 (27.57-78.28)

Southeast Asia	Dietary iron deficiency	<5 years	232091 (147059-355886)	425.97 (269.91-653.18)
Southeast Asia	Dietary iron deficiency	10-14 years	109670 (62200-170625)	190.64 (108.12-296.6)
Southeast Asia	Dietary iron deficiency	5-9 years	227869 (142248-351603)	401.45 (250.61-619.44)
Southeast Asia	Iodine deficiency	<5 years	99 (40-194)	0.18 (0.07-0.36)
Southeast Asia	Iodine deficiency	10-14 years	2147 (1081-3751)	3.73 (1.88-6.52)
Southeast Asia	Iodine deficiency	5-9 years	658 (271-1287)	1.16 (0.48-2.27)
Southeast Asia	Protein-energy malnutrition	<5 years	327709 (258602-404016)	601.47 (474.63-741.52)
Southeast Asia	Protein-energy malnutrition	10-14 years	25973 (18398-36179)	45.15 (31.98-62.89)
Southeast Asia	Protein-energy malnutrition	5-9 years	42023 (32426-54316)	74.03 (57.13-95.69)
Southeast Asia	Vitamin A deficiency	<5 years	27669 (17700-42302)	50.78 (32.49-77.64)
Southeast Asia	Vitamin A deficiency	10-14 years	8684 (5236-13323)	15.09 (9.1-23.16)
Southeast Asia	Vitamin A deficiency	5-9 years	17610 (10699-26802)	31.02 (18.85-47.22)
Southern Latin America	Dietary iron deficiency	<5 years	18964 (10863-32414)	390.66 (223.79-667.73)
Southern Latin America	Dietary iron deficiency	10-14 years	3388 (1223-7855)	68.68 (24.8-159.23)
Southern Latin America	Dietary iron deficiency	5-9 years	9568 (3583-19936)	186.93 (69.99-389.48)
Southern Latin America	Iodine deficiency	<5 years	5 (2-11)	0.1 (0.03-0.22)
Southern Latin America	Iodine deficiency	10-14 years	73 (28-148)	1.48 (0.57-3.01)
Southern Latin America	Iodine deficiency	5-9 years	33 (12-68)	0.64 (0.24-1.33)
Southern Latin America	Protein-energy malnutrition	<5 years	5449 (4163-7026)	112.25 (85.75-144.74)
Southern Latin America	Protein-energy malnutrition	10-14 years	683 (470-981)	13.84 (9.54-19.89)
Southern Latin America	Protein-energy malnutrition	5-9 years	839 (588-1168)	16.4 (11.48-22.83)
Southern Latin America	Vitamin A deficiency	<5 years	1323 (687-2439)	27.25 (14.14-50.25)
Southern Latin America	Vitamin A deficiency	10-14 years	180 (48-494)	3.66 (0.98-10.01)
Southern Latin America	Vitamin A deficiency	5-9 years	631 (213-1387)	12.33 (4.17-27.09)
Southern Sub-Saharan Africa	Dietary iron deficiency	<5 years	35712 (22005-54712)	441.09 (271.79-675.76)
Southern Sub-Saharan Africa	Dietary iron deficiency	10-14 years	27587 (14953-43742)	367.15 (199.01-582.16)

Southern Sub-Saharan Africa	Dietary iron deficiency	5-9 years	43336 (25431-68074)	541.42 (317.72-850.48)
Southern Sub-Saharan Africa	Iodine deficiency	<5 years	42 (17-88)	0.52 (0.21-1.09)
Southern Sub-Saharan Africa	Iodine deficiency	10-14 years	600 (249-1220)	7.98 (3.31-16.24)
Southern Sub-Saharan Africa	Iodine deficiency	5-9 years	269 (112-566)	3.37 (1.4-7.07)
Southern Sub-Saharan Africa	Protein-energy malnutrition	<5 years	289582 (207513-386760)	3576.69 (2563.05-4776.97)
Southern Sub-Saharan Africa	Protein-energy malnutrition	10-14 years	3846 (2625-5334)	51.19 (34.94-70.99)
Southern Sub-Saharan Africa	Protein-energy malnutrition	5-9 years	13843 (9993-18157)	172.94 (124.84-226.85)
Southern Sub-Saharan Africa	Vitamin A deficiency	<5 years	4835 (3015-7480)	59.72 (37.24-92.38)
Southern Sub-Saharan Africa	Vitamin A deficiency	10-14 years	1936 (1155-2969)	25.77 (15.37-39.52)
Southern Sub-Saharan Africa	Vitamin A deficiency	5-9 years	3668 (2258-5682)	45.82 (28.21-70.99)
Tropical Latin America	Dietary iron deficiency	<5 years	75584 (43782-124469)	468.52 (271.39-771.54)
Tropical Latin America	Dietary iron deficiency	10-14 years	37359 (15462-69576)	220.07 (91.08-409.85)
Tropical Latin America	Dietary iron deficiency	5-9 years	69277 (34469-123491)	417.22 (207.59-743.73)
Tropical Latin America	Iodine deficiency	<5 years	9 (3-19)	0.06 (0.02-0.12)
Tropical Latin America	Iodine deficiency	10-14 years	143 (55-305)	0.84 (0.33-1.8)
Tropical Latin America	Iodine deficiency	5-9 years	60 (22-127)	0.36 (0.13-0.76)
Tropical Latin America	Protein-energy malnutrition	<5 years	88998 (70561-110539)	551.67 (437.38-685.19)
Tropical Latin America	Protein-energy malnutrition	10-14 years	4343 (3483-5498)	25.58 (20.52-32.39)
Tropical Latin America	Protein-energy malnutrition	5-9 years	5178 (4110-6531)	31.19 (24.76-39.34)
Tropical Latin America	Vitamin A deficiency	<5 years	5695 (3372-9144)	35.3 (20.9-56.68)
Tropical Latin America	Vitamin A deficiency	10-14 years	3108 (1600-5346)	18.31 (9.42-31.49)
Tropical Latin America	Vitamin A deficiency	5-9 years	5396 (2767-9518)	32.5 (16.67-57.32)
Western Europe	Dietary iron deficiency	<5 years	23496 (14105-37451)	106.81 (64.12-170.25)
Western Europe	Dietary iron deficiency	10-14 years	6372 (3302-11227)	26.98 (13.98-47.53)
Western Europe	Dietary iron deficiency	5-9 years	22277 (12131-37777)	96 (52.28-162.79)
Western Europe	Iodine deficiency	<5 years	58 (22-123)	0.26 (0.1-0.56)

Western Europe	Iodine deficiency	10-14 years	1079 (427-2194)	4.57 (1.81-9.29)
Western Europe	Iodine deficiency	5-9 years	405 (156-856)	1.75 (0.67-3.69)
Western Europe	Protein-energy malnutrition	<5 years	595 (485-723)	2.7 (2.2-3.29)
Western Europe	Protein-energy malnutrition	10-14 years	9871 (5618-15993)	41.79 (23.79-67.72)
Western Europe	Protein-energy malnutrition	5-9 years	10681 (5781-17211)	46.03 (24.91-74.17)
Western Europe	Vitamin A deficiency	<5 years	343 (192-557)	1.56 (0.87-2.53)
Western Europe	Vitamin A deficiency	10-14 years	51 (23-96)	0.21 (0.1-0.41)
Western Europe	Vitamin A deficiency	5-9 years	279 (142-509)	1.2 (0.61-2.19)
Western Sub-Saharan Africa	Dietary iron deficiency	<5 years	1139710 (742425-1645060)	1567 (1020.77-2261.81)
Western Sub-Saharan Africa	Dietary iron deficiency	10-14 years	523811 (319794-793211)	882.15 (538.56-1335.85)
Western Sub-Saharan Africa	Dietary iron deficiency	5-9 years	1184334 (781181-1749036)	1788.48 (1179.68-2641.25)
Western Sub-Saharan Africa	Iodine deficiency	<5 years	567 (261-1057)	0.78 (0.36-1.45)
Western Sub-Saharan Africa	Iodine deficiency	10-14 years	7162 (3590-12844)	12.06 (6.05-21.63)
Western Sub-Saharan Africa	Iodine deficiency	5-9 years	3497 (1611-6398)	5.28 (2.43-9.66)
Western Sub-Saharan Africa	Protein-energy malnutrition	<5 years	2744455 (2010115-3648427)	3773.39 (2763.73-5016.27)
Western Sub-Saharan Africa	Protein-energy malnutrition	10-14 years	31190 (22369-41879)	52.53 (37.67-70.53)
Western Sub-Saharan Africa	Protein-energy malnutrition	5-9 years	83665 (58663-115587)	126.34 (88.59-174.55)
Western Sub-Saharan Africa	Vitamin A deficiency	<5 years	114156 (78333-162171)	156.95 (107.7-222.97)
Western Sub-Saharan Africa	Vitamin A deficiency	10-14 years	42228 (27629-60286)	71.12 (46.53-101.53)
Western Sub-Saharan Africa	Vitamin A deficiency	5-9 years	80154 (53004-113886)	121.04 (80.04-171.98)
